# The role of subcortical brain tissue iron as an indicator of dopamine neurophysiology in adolescent cannabis use

**DOI:** 10.1038/s41386-026-02444-9

**Published:** 2026-06-02

**Authors:** Sarah A. Thomas, Meghan A. Gonsalves, Gillian LeBlanc, Elizabeth Lorenc, Jane Metrik, Michael Frank, Sarah Ryan, Emily Olenik, Leslie Brick, Anthony Spirito, Jodi Gilman

**Affiliations:** 1 Bradley Hasbro Children’s Research Center, Providence, RI USA; 2 Alpert Medical School of Brown University Department of Psychiatry and Human Behavior, Providence, RI USA; 3 Carney Institute for Brain Science, Brown University, Providence, RI USA; 4 Center for Alcohol and Addiction Studies, Brown University School of Public Health, Providence, RI USA; 5 Brown University Dept. of Neuroscience, Providence, RI USA; 6 Providence VA Medical Center, Providence, RI USA; 7 Brown University Department of Cognitive and Psychological Sciences, Providence, RI USA; 8 Center on Alcohol, Substance Use, and Addiction, University of New Mexico, Albuquerque, NM USA; 9 Massachusetts General Hospital (MGH) Department of Psychiatry, Boston, MA USA; 10 Harvard Medical School, Boston, MA USA

**Keywords:** Reward, Human behaviour, Addiction

## Abstract

Approximately 10–20% of U.S. adolescents report past-year cannabis use (CU). Although regular CU beginning in adolescence is expected to blunt dopamine-related neurophysiology, this hypothesis has not been tested in adolescents due to methodological limitations. However, neurophysiology contributing to dopamine can be noninvasively indexed via subcortical tissue iron measured with magnetic resonance imaging (MRI). We examined adolescent CU quantity, frequency, and problems in relation to tissue iron in regions with high dopamine activity, hypothesizing that greater CU would be linked to less tissue iron. Adolescents (*n* = 81; 64.2% female) aged 14–17 reporting either fewer than 5 lifetime cannabis episodes (*n* = 47) or more than 11 episodes (*n* = 34), with limited alcohol and nicotine use and no other illicit substance use, completed substance use assessments and an MRI. We calculated the inverse of the normalized T2* measurement (1/nT2*; lower values indicate less tissue iron) from resting-state functional scans by assessing relative T2* decay. 1/nT2* was estimated using subcortical masks for hypothesized regions. Lower 1/nT2* signal was associated with increased daily concentrate hits (*b* = –0.01, *p* < 0.001), cannabis hours high (*b* = –0.01, *p* = 0.016), CU frequency (*b* = -0.01, *p* = 0.01), and cannabis use disorder (CUD) severity (*b* = –0.01, *p* = 0.003). Post-hoc analyses highlighted the VTA as a key region. Results align with reduced dopamine-related neurophysiology associated with CU in adult and animal samples, and have implications for understanding adolescent CUD development. Measuring 1/nT2* offers an innovative, non-invasive method to index neurobiological alterations in adolescent CU.

## Introduction

Up to 21% of US adolescents ages 14–17 have used cannabis in the past year [1]. Studies in humans and animals provide strong evidence that adolescence is a vulnerable period to start cannabis use (CU) because it interferes with the brain’s dynamic development process [2–10]. In fact, CU during adolescence, compared to adulthood, is linked to a higher risk of developing cannabis use disorder (CUD) and experimenting with other drugs [11–13]. Greater risk of negative outcomes has been attributed to the role CU plays in neuroadaptations during adolescence [14], especially the dopaminergic system [15, 16], which is the focus of the current study.

Brain development is supported by the endogenous cannabinoid system (ECS) [8, 17–20] and may be hindered by exogenous CU during adolescence [2, 9, 18, 21, 22]. When cannabis activates the ECS, it also impacts other biological systems, including the modulation of neurotransmitters like dopamine, which has been closely linked to rewarding experiences and motivation [23–26]. Dopamine also plays a key role in drug addiction [27–29]. It is produced by tyrosine hydroxylase, an iron-dependent enzyme, and aromatic L-amino acid decarboxylase, and is stored in vesicles by the vesicular monoamine transporter [30, 31]. Generally, the rewarding effects of acute drug intoxication are attributed to increased dopamine in the striatum [28, 32–34] originating from dopamine neurons in the ventral tegmental area (VTA) [27, 35]. In contrast, habitual drug use over time is linked to decreased dopaminergic activity, such as reduced dopamine receptor availability in the striatum [36–41].

Knowledge of how cannabis affects the brain, particularly the dopaminergic system, remains incomplete [42, 43], especially among adolescents. In adults, acute tetrahydrocannabinol (THC) administration increases dopamine release [34]. Chronic CU is expected to have a blunting effect on the mesolimbic dopamine system, leading to reduced synaptic transmission and decreased synthesis capacity [16, 43–45], as well as less dopamine release during a stimulant drug challenge in PET imaging [42, 46]. The link between chronic THC exposure and blunted dopamine synthesis is supported in both humans and rodents [24, 44, 47]. For example, adults with chronic CU who experienced psychotic symptoms had significantly lower striatal dopamine synthesis capacity compared to controls [47].

Dopaminergic indices are typically measured with PET imaging, which is limited for adolescent research due to radioactive exposure. Alternatively, tissue iron in subcortical brain regions is an indirect proxy for dopamine-related neurophysiology, indexed through the inverse-normalized T2*-weighted contrast (1/nT2*) [48–51]. Tissue iron co-localizes with dopamine vesicles and is essential for dopamine synthesis [30, 48, 52]. It is paramagnetic and most concentrated in the midbrain and basal ganglia [48], with higher levels significantly increasing the T2* relaxation rate. Therefore, single- and multi-echo magnetic resonance imaging (MRI) techniques that measure the T2* relaxation rate can assess tissue iron [50, 53, 54]. Furthermore, a significant positive correlation exists between multi-echo MRI measurements of tissue iron in the nucleus accumbens (NAcc) and PET-based measures of presynaptic vesicular dopamine storage [48]. It is important to note that while 1/nT2* substantially reflects iron content in high-iron concentration regions that contribute to catecholamine (including dopamine) synthesis, as well as oxidative and other neuronal processes [55], it may also reflect other tissue properties (e.g. myelin content [56]).

Tissue iron has been measured in relation to various cognitive domains, psychiatric conditions, and drug use linked to dopamine-related neurophysiology [50, 53, 57–61], and shows excellent reliability for within-session measurements [51]. Basal ganglia tissue iron levels increase with age during adolescent neurodevelopment [59, 62–65] and follow the same developmental curve from adolescence to adulthood as dopamine-related neurophysiology [48, 59]. The main factor affecting changes in T2* relaxation rates is ferric iron [66], making this proxy measure of tissue iron a valuable indicator of processes and activities requiring iron, including regional dopamine function.

Repeated CU is believed to cause neurobiological adaptations over time that lead to problematic changes in behavior, cognition, emotion, and CUD [67–70]. Assessing dopamine-related neurophysiology is a crucial first step in evaluating the link between exogenous CU and the developing adolescent brain. Although the dopaminergic system undergoes significant development during adolescence [16, 71], dopamine-related neurophysiology has not been studied in relation to CU in adolescents. Additionally, with the dramatic increase in cannabis potency [72, 73], and adolescents increasingly preferring concentrated products [74–76], previous research on CU may be outdated in the context of high-potency products used now. Higher THC potency is associated with a faster progression to the first CUD symptom [77] and the development or worsening of mental health conditions [78, 79]. Since youth generally have less exposure to other illicit drugs, it is somewhat easier to interpret this relationship. This study aims to address key research gaps regarding the connection between repeated adolescent CU and dopamine-related neurophysiology. We measured CU quantity, frequency, and CUD symptom severity, along with tissue iron levels in key subcortical regions with high dopamine activity in 14–17-year-olds. We hypothesized an inverse relationship between CU and tissue iron, consistent with previous research indicating a link between CU and reduced dopamine synthesis capacity [16, 24, 44, 45, 47].

## Methods

### Participants

Participants were recruited through community flyers, local schools, social media advertisements, and review of electronic medical records at the affiliated hospital. The study received approval from E.P. Bradley Hospital’s Institutional Review Board and required caregiver consent and adolescent assent. Participants met the following inclusion criteria: (a) ages 14–17; (b) reported either <5 lifetime cannabis episodes (“control group”) or >11 episodes (“cannabis group”); (c) no history of head injury with loss of consciousness >10 min, seizures, or migraines based on caregiver report; (d) no current impairing psychosis; and (e) ability to abstain from cannabis for 15 hours without withdrawal. Eligibility criteria aimed to (1) maximize group differences to capture established, non-experimental CU; (2) isolate cannabis-related neurobiological variations by limiting exposure to nicotine, alcohol, and other illicit substances, which have independently been linked to dopaminergic and subcortical changes in prior neuroimaging studies [60, 61, 80–82]; (3) facilitate recruitment—a high CU rate might co-occur with other (excluded) drug use; and (4) account for the reduction in episodes observed in recent years, possibly due to adolescents favoring vaping products [74, 83]. Since both high-potency product use and adolescence increase the rapid risk of CUD [77, 84], adolescents often reported much greater CU than the inclusion threshold. Adolescents were ineligible if: (a) their past 3-month alcohol use was at least weekly and averaged to >4 drinks per episode or >4 drinks per episode more than once a month [85]; (b) they scored “high dependence” (>12) on the Electronic Cigarette Dependence Index [86] or reported using other nicotine products at ≥3 times per week over the past 3 months; or (c) reported any other lifetime substance use. Five female control participants completed all procedures during a pilot phase with slightly different procedures, noted below. An additional seventy-eight participants completed all procedures. Two participants with poor-quality functional MRI data (described below) were excluded from analyses, resulting in a final sample of 81 adolescents. Table 1 presents demographics and substance use details by group.

**Table 1:** Summary of sample demographics and substance use patterns.

Variable	*N*	Control *N* = 47	Cannabis *N* = 34	Statistic	*p* value
**Age, Mean (SD)**	81	15.34 (1.01)	15.79 (1.23)	–1.77	0.082
**Biological Sex, n (%)**	81				
Female		30 (64%)	22 (65%)		
Male		17 (36%)	12 (35%)		
**Race, n (%)**	81				
Asian		1 (2.1%)	1 (2.9%)		
Biracial		2 (4.3%)	5 (15%)		
Black		3 (6.4%)	5 (15%)		
Other		1 (2.1%)	3 (8.8%)		
Unknown		1 (2.1%)	0 (0%)		
White		39 (83%)	20 (59%)		
**Hispanic, n (%)**	81				
No		39 (83%)	18 (52.9%)		
Yes		6 (13%)	16 (47.1%)		
Do not know		1 (2.1%)	0 (0%)		
Unknown		1 (2.1%)	0 (0%)		
**Medication at MRI**					
Anti-psychotic, n (%)	81	1 (2.1%)	2 (5.9%)	2.84	0.569
Antidepressant, n (%)	81	6 (13%)	11 (32%)	3.22	0.051
Stimulant, n (%)	81	1 (2.1%)	1 (2.9%)	1.39	1.00
Sleep, n (%)	81	2 (4.3%)	3 (8.8%)	2.16	0.645
Non-stimulant, n (%)	81	1 (2.1%)	0 (0%)	0.00	1.00
**Substance Use, Mean (SD)**					
Daily grams of cannabis	81	0.01 (0.07)	0.87 (1.27)	–3.93	<0.001
Daily hits of cannabis concentrates	79	0.00 (0.00)	10.97 (8.94)	–6.94	<0.001
TLFB-Average daily hours high	81	0.01 (0.03)	1.91 (2.95)	–3.76	0.001
TLFB-Average daily cannabis sessions	81	0.00 (0.01)	1.05 (1.31)	–4.64	<0.001
TLFB % cannabis concentrate days	81	0.00 (0.00)	0.23 (0.28)	–4.81	<0.001
TLFB % cannabis flower days	81	0.00 (0.00)	0.21 (0.33)	–3.72	0.001
TLFB % cannabis edible days	81	0.00 (0.01)	0.01 (0.01)	–1.49	0.14
TLFB % alcohol days	81	0.01 (0.02)	0.02 (0.02)	–2.53	0.014
TLFB % nicotine days	81	0.00 (0.01)	0.24 (0.38)	–3.75	0.001
**Current CUD Diagnostic Status, n (%)**	81				
Not present		47 (100%)	4 (12%)		
Mild		0 (0%)	7 (21%)		
Moderate		0 (0%)	12 (35%)		
Severe		0 (0%)	11 (32%)		

Welch’s *t* tests for continuous variables, Fischer’s exact test for medication group differences. *CUD* cannabis use disorder.

### Procedures

At baseline, eligible participants and their caregivers completed the consent/assent process, surveys, a brief intelligence screening, Timeline Follow-Back Interview (TLFB) [87], semi-quantitative urine screen, and a behavioral task (not reported here). The average number of days between the baseline and MRI visits was 19.49 (SD = 11.57) for the entire sample, except for two pilot participants whose MRI visits were delayed due to the COVID-19 pandemic (naïve to all substances at baseline and subsequent MRI). Adolescents were instructed to abstain from all substance use for 15 hours before the MRI. At the MRI visit, adolescents completed a mock scanner practice where they listened to MRI sounds in a magnet-free MRI simulator to reduce anxiety. They also completed a saliva drug screen and a TLFB to record substance use since the baseline visit. The MRI lasted 60 min and included structural and functional scans (tasks, resting state). Afterward, adolescents completed surveys on substance use correlates and a CUD diagnostic interview. At both visits, caregivers reported adolescents’ current medications (Table S1).

### Measures

#### Weschler Abbreviated Scale of Intelligence II (WASI-II)

The WASI-II [88] was administered by trained research staff to determine research-based two sub-test (vocabulary; matrix reasoning) full-scale IQ for eligibility. Participants scoring below 80 were ineligible (*n* = 2) and did not continue study procedures.

#### Daily Sessions, Frequency, Age of Onset, and Quantity of Cannabis Use Inventory (DFAQ-CU) [89, 90]

This self-report assessed typical cannabis quantity used, method (e.g., vaping, edibles), and patterns of administration at baseline. Of relevance are the following questions that assess the past 3 months: “On a typical day you use marijuana, how much do you personally use?” which refers adolescents to an image of flower quantities to estimate in grams, and “On a typical day when you use cannabis concentrates, how many hits do you personally take?”

#### Timeline Follow-Back Interview (TLFB) [87]

A calendar-based TLFB interview was completed for the 90 days before the baseline visit to assess number of cannabis sessions, formulation (flower, concentrates, and/or edibles), and subjective intoxication (“hours high”). Participants also reported on daily alcohol use (standard drinks) and use of tobacco or nicotine (yes/no). At the MRI visit, participants completed the TLFB for the days since the baseline visit to obtain a comprehensive assessment of recent substance use. Because the measurement periods varied (90 days plus time between the baseline and MRI visits), relevant TLFB variables were calculated as rates of use (cannabis sessions, drinks per day) and proportion of use days (concentrate or flower days) by dividing the substance use value by the total number of measurement days. During the MRI TLFB, four teens acknowledged day-of-MRI nicotine use, but all had negative THC saliva drug tests (described below). Adolescents reported minimal use of cannabis edibles, so this formulation was not explicitly tested.

Additional questions included in the interview were on lifetime quantity or episodes of tobacco/nicotine products, alcohol (number of standard drinks), and cannabis (grams for flower and number of times concentrates were used) [91], as well as one question each on the potency (i.e., % THC) of the smoked flower and concentrated cannabis used in the past six months.

#### CUD

Adolescents in the cannabis group completed the Kiddie Schedule for Affective Disorders and Schizophrenia (K-SADS) CUD module interview by a licensed clinical psychologist to determine the presence and severity of current (past 12 months) and past CUD. CUD symptom severity was coded as 0 for ≤1 symptom (the score control group teens received), 1 for mild (2–3 symptoms), 2 for moderate (4–5 symptoms), and 3 for severe CUD (≥ 6 symptoms).

#### Drug screens

Participants completed two drug screens during the study. At the baseline visit, a Narcocheck semi-quantitative urine screen measured six possible THC levels: not positive (59.26% of the sample), level 1 (18 ng/ml; 7.41%), level 2 (50 ng/ml; 1.23%), level 3 (150 ng/ml; 6.17%), level 4 (300 ng/ml; 11.11%), and level 5 (600 ng/ml; 1.23%). We did not require a positive urine drug screen for enrollment eligibility. A total of 13.58% of the sample (*n* = 11) lacked test results: three adolescents declined the test; the urine test was not part of the pilot phase procedures (*n* = 5); and we did not administer the test to *n* = 3 control adolescents at the start of the study, as procedures were being established. The semi-quantitative urine screen results correlated significantly with self-reports of daily grams of smoked cannabis, hits of concentrated cannabis, and TLFB-derived indices of daily hours high, daily cannabis sessions, proportion of concentrate and flower days, and CUD severity (*r*s between 0.64 and 0.82, all *p* < 0.001).

At the MRI visit, participants completed a Narcocheck 5-panel saliva drug screen for THC, cocaine, opiates, amphetamines, and methamphetamines. No adolescents tested positive for cannabis or other drugs during the MRI; however, there were missing test results due to equipment issues (see Supplement). Participants with invalid tests denied recent substance use and underwent scanning.

#### Psychotic-like experiences (PLEs)

Participants completed the Achenbach Youth Self-Report (YSR), which derives empirically-based syndrome scales [92]. The Thought Problems 12-item subscale assesses PLEs (e.g., hallucinations, delusions); its t-score was used as a covariate.

### MRI acquisition

Data were acquired on a Siemens 3 T PRISMA scanner using a 64-channel head coil. An MPRAGE T1 structural scan was acquired with the following parameters: TR/TE/TI = 1900 ms/3.02 ms/ 900 ms, flip angle = 9°, FOV = 256 mm, 160 slices, voxel size = 1 mm^3^. T2*-weighted, single-shot, gradient-echo, echo-planar imaging (EPI) resting state sequences, in which participants were instructed to relax with their eyes open were 9 min and acquired with the following parameters for the pilot participants: TR = 2000 ms, TE = 30 ms; flip angle = 90°; FOV = 192 mm; 60 slices; 256 measurements; voxel size 2mm^3^; multiband acceleration factor 3; GRAPPA in-plane acceleration factor 2, and for the remaining participants (*n* = 76) TR = 1600 ms, TE = 30 ms; flip angle = 62°; FOV = 200 mm; 72 slices; 333 measurements; voxel size 2 mm^3^; multiband acceleration factor 3; GRAPPA in-plane acceleration factor 2. In all but the initial 5 pilot participants, to improve orbitofrontal signal, Siemens AutoAlign was used to tilt the slice prescription 30 degrees relative to the AC-PC line [93]. Differences in the two protocols were due to the tilt and sequence optimization (additional slices and slightly larger field of view to improve coverage, and reduced TR to acquire more samples in the same amount of time).

### MRI processing

We calculated the inverse of the normalized T2* measurement (1/nT2*) from EPI scans using the following steps. Because we acquired single-echo EPI versus multi-echo, we followed Sonnenschein and colleagues [50, 52] by calculating relative T2* decay using a single echo to derive tissue iron estimates in each voxel compared to the whole brain. This provides a single estimate of T2* decay for each voxel, yielding a whole-brain map of this proxy measure of tissue iron.

Functional and anatomical data were preprocessed with the CONN Toolbox (version 22a) supported by MATLAB (version R2023a; Natick, MA, The MathWorks, Inc.) using a flexible, standard preprocessing pipeline [94] (see Supplement). Participants were included in subsequent analyses if their scans contained at least 170 volumes [53] after excluding any volumes with 0.5 mm movement [50]. Two participants were excluded at this stage due to having fewer than 170 valid volumes.

**Fig. 1:**
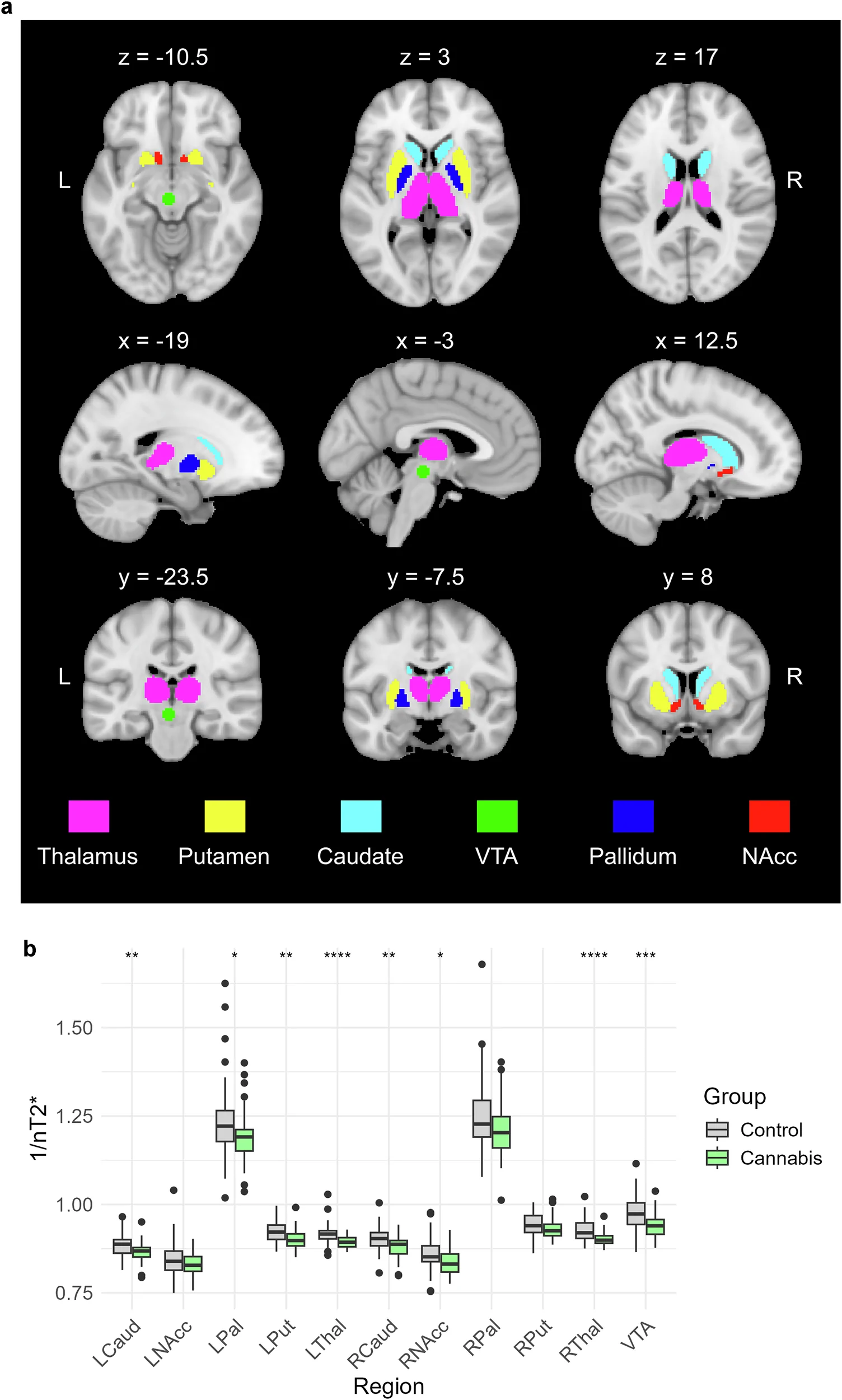
Characteristics of regions in which 1/nT2* was measured. **a** Regions in which 1/nT2* was evaluated due to expectation of dopamine presence. NAcc nucleus accumbens, VTA ventral tegmental area. **b** Boxplot depicting bivariate group differences in subcortical 1/nT2*. VTA ventral tegmental area, LNAcc left nucleus accumbens, RNAcc right nucleus accumbens, LCaud left caudate, RCaud right caudate, LPut left putamen, RPut right putamen, LThal left thalamus, RThal right thalamus, LPal left pallidum, RPal right pallidum. * *p* < 0.05; ** *p* < 0.01; *** *p* < 0.001.

To derive 1/nT2* estimates across the whole brain, this preprocessed functional data was processed in AFNI to a 1-volume output in which each voxel is the inverse of the normalized T2* measurement (1/nT2*) [51]. This was done by: (1) normalizing the voxels of each volume (timepoint) to the mean of that volume within an MNI-template brain mask; (2) taking the median of each voxel’s normalized intensity across time; and (3) calculating the inverse of this value. The output is a single-volume 1/nT2* map for each participant. Measurements are presented as the inverse to simplify interpretation, as the T2*-weighted signal is inversely related to tissue iron concentration. Consequently, lower 1/nT2* values indicate less tissue iron. Subcortical regions implicated in reward and addiction and where dopamine concentrations were expected to be the highest were chosen as regions of interest (ROIs; Fig. 1A) [27, 35, 48, 50, 51, 62, 71, 95]. The mean 1/nT2* value was calculated for each ROI using AFNI’s 3dmaskave. Masks derived per-participant 1/nT2* levels for each of the ROIs: caudate, putamen, NAcc, pallidum, and thalamus were made in FSL [96] (version 6.0.7.7) using the Harvard-Oxford atlas, while the VTA was a 5 mm radius sphere [97, 98] centered at MNI -2, -22, -12. We selected this conservative ROI to minimize the impact of partial volume effects—proportion of CSF voxels: 0.03 (SD = 0.02), white matter: 0.84 (SD = 0.07), gray matter 0.13 (SD = 0.07). Each participant had 100% EPI slice coverage of the VTA ROI sphere (and sufficient signal in every voxel to survive brain extraction with AFNI’s 3dAutomask). The average temporal signal-to-noise ratio within the VTA was 50.58 (SD = 8.41). CONN’s motion correction procedure minimized the impact of distortion because it reduced any potential interactions between susceptibility distortion and motion by resampling each EPI volume to match the deformation field of the reference functional image [94]. Without fieldmaps, we were unable to apply fieldmap-based susceptibility distortion correction, although our use of GRAPPA (acceleration factor = 2) reduced the magnitude of in-plane susceptibility distortion [99]. Finally, using AutoAlign to achieve a consistent 30 degree tilt relative to the AC-PC line did not impact midbrain/VTA coverage.

### Statistical analyses

All substance use variables reported in the analyses except CUD were skewed and were log-transformed before including in models. All models controlled for age, sex, self-reported nicotine and alcohol use, and PLEs [100] due to their documented association with dopamine-related neurophysiology. Three pilot control adolescents lacked PLE data and were excluded from the mixed models described below. Age is linked to increased tissue iron concentration in the basal ganglia during adolescence [48, 49], and was positively correlated with pallidum 1/nT2* (*r* = 0.27, *p* = 0.016). Biological sex was negatively correlated with VTA 1/nT2* (*r* = –0.34, *p* = 0.002). Psychosis was recently found to be related to CU and dopamine-related neurophysiology [100]. Alcohol and/or nicotine use, which may also be related to dopamine-related neurophysiology [101–104], match the same timeframe as CU in the models.

Analyses were performed in R version 4.4.1 [105–111]. Using the R package lme4 [112], main analyses consisted of five separate linear mixed models to examine the relationships between CU patterns (quantity of daily smoked cannabis in grams and concentrate hits, frequency defined as cannabis session rate during TLFB measurement, daily hours high, and CUD symptom severity) as the independent variables and subcortical 1/nT2* signal as the dependent variable. To minimize multiple comparisons, brain region was treated as a repeated measure (11 regions). One random effect allowed the intercept (1/nT2*) to vary by brain region; another random effect allowed the intercept to vary by adolescent. Significant associations were followed by post hoc linear regression analyses, which included the same covariates as the mixed models, to identify significant regions (see Supplement). The Supplement describes the analytic plan for additional sensitivity and post hoc follow-up analyses.

## Results

**Fig. 2:**
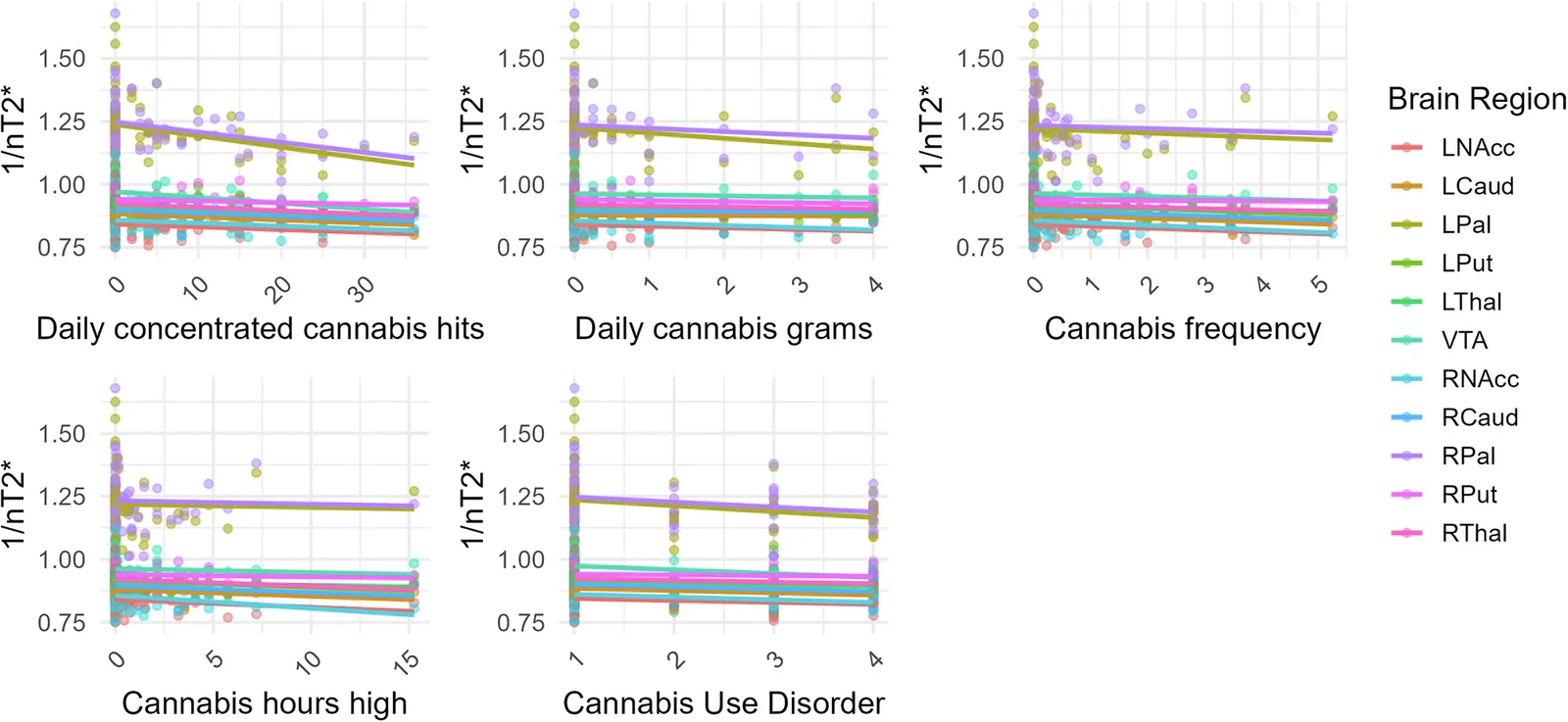
Scatterplots depicting associations between cannabis use measures and subcortical 1/nT2*. Cannabis use variables were not log-transformed. Frequency = cannabis use sessions; Current cannabis use disorder severity (coded as 1 = none, 2 = mild, 3 = moderate, 4 = severe). VTA ventral tegmental area, LNAcc left nucleus accumbens, RNAcc right nucleus accumbens, LCaud left caudate, RCaud right caudate, LPut left putamen, RPut right putamen, LThal left thalamus, RThal right thalamus, LPal left pallidum, RPal right pallidum.

In many brain regions, adolescents in the cannabis group had significantly lower 1/nT2* levels than controls (Fig. 1B; see Supplementary Table S2). The relationship between cannabis measure and 1/nT2* by brain region is depicted continuously with scatterplots in Fig. 2. The pallidum exhibited the highest 1/nT2* signal, consistent with previous studies [50, 52, 63, 71].

Among adolescents with any lifetime CU, 24.39% reported starting between ages 8-12, 21.95% at age 13, 19.51% at age 14, 17.07% at age 15, 12.20% at age 16, and 4.88% at age 17. In the cannabis group, teens reported 21% of flower days and 23% of concentrate days (not mutually exclusive). When asked “how strong or potent do you think your smoked marijuana was that you used in the past 6 months”, 3.45% said it was low (around <5% THC); 10.34% said medium (10% THC); 31.03% said high (15% THC); 27.59% said very high (20% + THC); and 27.59% did not know. Regarding cannabis concentrates, in response to “how strong or potent do you think your typical marijuana concentrate was in the past 6 months”, 5.71% responded low (around 20% THC); 25.71% medium (40% THC); 17.14% high (60% THC); 14.29% very high (80% + THC); and 37.14% did not know.

More daily hits of cannabis concentrates were significantly associated with lower 1/nT2* (Table 2, Model 1). Post-hoc regressions showed that more daily concentrate hits were significantly linked to lower 1/nT2* signal in the left and right pallidum, left and right thalamus, and VTA (Table S3). Self-reported daily grams of cannabis flower were not significantly related to 1/nT2* signal (Table 2, Model 2). Greater daily hours high on the TLFB were also significantly associated with lower overall subcortical 1/nT2* signal (Table 2, Model 3); post-hoc regressions did not identify specific regions with lower 1/nT2* (Table S4).

**Table 2:** The association between cannabis quantity (models 1 and 2), subjective intoxication (model 3), cannabis frequency (model 4), and cannabis use disorder severity (model 5) with subcortical 1/nT2* signal intensity.

	Model 1 (*n* = 76)			Model 2 (*n *= 78)			Model 3 (*n* = 78)		
*Predictors*	*Estimates*	*CI (95%)*	*p*	*Estimates*	*CI (95%)*	*p*	*Estimates*	*CI (95%)*	*p*
Intercept	0.87	0.73 – 1.01	<0.001	0.83	0.69 – 0.98	<0.001	0.84	0.70–0.98	<0.001
Daily concentrate hits (log)	–0.01	–0.02 – <–0.01	<0.001						
Daily cannabis grams (log)				–0.01	–0.02 to 0.00	0.094			
Daily hours high ratio (log)							–0.01	–0.02 to <–0.01	0.016
		**Model 4** (***n*** = **78**)			**Model 5** (***n*** = **78**)				
Intercept	0.84	0.70–0.99	<0.001	0.86	0.72–1.00	<0.001			
Cannabis sessions ratio (log)	–0.01	–0.02 to <–0.01	0.010						
CUD severity				–0.01	–0.02 to <–0.01	0.003			

Each model was run separately.

*CI* confidence interval, *PLE* psychotic-like experiences, *CUD* cannabis use disorder.

More CU sessions were linked to significantly lower 1/nT2* values overall (Table 2, Model 4). Post-hoc follow-up regressions implicated the left pallidum and left putamen (Table S5). Lastly, greater CUD symptom severity was associated with lower 1/nT2* signal (Table 2, Model 5), with post-hoc regressions implicating the left and right pallidum and VTA (Table S6).

### Sensitivity analyses

Sensitivity analyses are included in the Supplement. The same pattern of results remained when adjusting for scan protocol, medication, and nicotine use on the MRI day. Exploratory follow-up results are also included in the Supplement. We highlight that concentrate days, but not flower days, were a significant predictor of lower 1/nT2* values, and that excluding participants with nicotine use during the TLFB measurement period produces a similar pattern as the main analyses, with some additional significant findings.

## Discussion

This is the first study to examine the relationship between CU and an estimate of tissue iron, an indirect contribution to dopamine-related neurophysiology in adolescent humans. We evaluated whether there was an inverse association between CU and subcortical 1/nT2*, a marker for tissue iron, among 14–17-year-old adolescents. Results showed that cannabis quantity, frequency, hours high, and CUD severity were significantly negatively associated with subcortical 1/nT2* signal, even after controlling for age, biological sex, nicotine, and alcohol. The study’s design, measuring CU and 1/nT2* simultaneously during adolescence rather than relying on retrospective recall in adulthood, and adolescents having no other illicit drug use besides CU, nicotine, and alcohol, enhances the methodological strength of our findings.

Our findings support the idea that repeated CU is associated with reduced dopamine-related neurophysiology [15, 16, 42, 44]. Studies on adults with CU have shown mixed results regarding dopamine-related neurophysiology, depending on which PET-derived index was measured (reviewed here [43, 45, 82]). That CU might specifically impact dopamine synthesis is increasingly supported as a possible explanation for how cannabis influences reward processing and motivation [113]. Consistent with our results, adults with CU exhibited significantly lower dopamine-related neurophysiology compared to those without CU, with a clear inverse relationship indicating that higher CU was associated with lower striatal synthesis capacity [47]. Although we used a different, indirect method to assess dopamine-related neurophysiology than typical adult CU studies, one important aspect of tissue iron levels is their connection to the critical role of iron in dopamine synthesis, particularly in presynaptic vesicular dopamine storage [48]. Explanations for why indicators of dopamine-related neurophysiology might be lower in CU than in control participants include fewer presynaptic dopamine vesicles available for release due to loss of dopamine terminals during repeated substance use, or reduced glutaminergic stimulation of dopamine neurons in the VTA [27, 114]. Alternatively, differences in dopamine-related neurophysiology may pre-date CU onset [115]. We emphasize that our interpretations are based on converging evidence from previous animal, postmortem, and PET studies [42–45, 47, 66, 116–118] linking iron availability to dopamine synthesis and storage, rather than direct measurement of dopamine function. However, iron contributes to more processes than just dopamine [118, 119], so the observed relationship could also involve other neurobiological mechanisms.

We observed the VTA and thalamus as frequently affected regions, with lower 1/nT2* signal associated with adolescent CU patterns. The VTA is a key part of the mesolimbic dopamine pathway and the dopamine motive system, both vital for reinforcement and goal-directed behaviors [70, 120]. These processes may be impaired with chronic drug use. In mice, repeated THC administration over three weeks led to decreased VTA dopamine activity, which the authors interpret as consistent with the idea that presynaptic dopamine hypofunction contributes to addiction risk [44]. The VTA plays an important role in neural adaptations associated with CU, starting with the ECS affecting dopamine signaling in the VTA, which then influences other striatal regions [43, 70, 121–123]. Our findings of lower 1/nT2* signal associated with CU in striatal areas (e.g., caudate, putamen) may indicate decreased motivation for non-drug rewards, potentially serving as a marker for future drug use risk as a compensatory response to dopamine deficits [11, 124], a central mechanism in addiction development [14, 15, 42, 67, 125]. This is supported by our finding that the VTA had significantly lower 1/nT2* signal with greater CUD severity. Lastly, the thalamus, a large, iron-rich region receiving dopaminergic projections and part of cortico-striatal circuitry, supports conditioned responses, neural adaptations, and incentive salience (“wanting”) [33, 70, 124, 126–129]. Its function and large size, less likely impacted by partial volume effects, may help explain our finding that individuals with daily concentrate hits showed lower bilateral 1/nT2* signal in the thalamus.

Our findings also indicate a connection between cannabis potency and dopamine-related neurophysiology. Although adolescents in this study reported they often did not know the potency of the cannabis they used, concentrate/oil products are likely to have significantly higher THC levels than flower material (e.g., >70% vs. 1–30%[130–132]), a difference reflected in plasma THC levels [133]. Concentrate use (both quantity and proportion of use days) appeared to have a stronger link to 1/nT2* signal than quantity or frequency of flower. Higher potency has been associated with more adverse effects and quicker onset of the first CUD symptom [77, 134, 135].

The current study has several strengths. Because dopamine-related neurophysiology increases during adolescence [48, 49, 52, 62, 136–141], this developmental stage is particularly important for examining its link to CU. Our TLFB assessed substance use three months before baseline and covered the period from then until the 1/nT2* measurement. Because adolescents in our study did not have a history of using any illicit substances besides cannabis, alcohol, or nicotine, we can rule out potential neural confounds from other drugs. Although we aimed to limit nicotine use among participants, CU often co-occurs with nicotine use [142–144]. Understanding the role of dopamine-related neurophysiology in CU has been complicated by this co-occurrence [43], and prior research over the past few decades has often not included nicotine as a covariate, which complicates interpreting the results [43]. We measured nicotine use and included it as a covariate to ensure that the significant relationships between CU characteristics and subcortical 1/nT2* signal remained. Although this study was not preregistered, hypotheses, ROIs, and analytic strategies were specified in advance based on existing theory and prior work[16, 47, 48, 50, 62, 145], and analytic flexibility was minimized through anatomically-defined ROIs and prespecified mixed-effects models.

Several study limitations warrant mention. Because adolescents could not have used other illicit drugs, we likely limited our sample to those with less chronic CU, since high-frequency and/or problematic use of one substance often co-occurs with other substance use [144, 146]. At the same time, the enrolled sample ultimately reflected predominantly frequent and higher-potency use, including high rates of CUD, despite the minimum cannabis exposure threshold being selected to include adolescents using approximately weekly over several months. Thus, results are most generalizable to adolescents with established, recurrent CU rather than light or experimental use. Relatedly, adolescents were unable to reliably report on the device type and cartridge-level potency of their vaped concentrated cannabis, so we could not derive the quantity of each hit. In legal markets, which includes where this study was conducted, concentrated vaping products have high THC concentration and are often used more frequently than flower[83, 147–149]. The cross-sectional design does not rule out that 1/nT2* signal differences preceded CU, and does not permit causal inference or prospective prediction. Because our imaging paradigm was not multi-echo EPI, the 1/nT2* metric should be interpreted as an indirect, susceptibility-sensitive proxy related to tissue iron rather than a quantitative measure of iron content [50]. Quantitative susceptibility mapping [150], which uses multi-echo, is a more direct, sensitive measure of tissue iron [58] that assesses magnetic susceptibility macroscopically and has a high contrast to distinguish it from other tissues [54, 151]. When combined with T2* decay indices [56, 63], it may be possible to disentangle other sources that contribute to the signal, including myelin (which has diamagnetic properties compared to tissue iron’s paramagnetic [59]). We also cannot rule out partial-volume effects influencing the VTA signal, despite caution in its selection [97, 98] and acquisition. Replication studies using higher-field, multi-echo, pre-registered protocols are planned and will be reported in future work. Therefore, findings should be interpreted as hypothesis-supporting rather than definitive.

To summarize, tissue iron is a critical factor in dopamine synthesis, which is the most frequently implicated dopamine-related neurophysiological process related to CU, as well as other tissue-based neurobiological processes. Considering the challenges of PET studies—cost, radiation exposure, and small sample sizes—measuring subcortical tissue iron in CU may be a practical alternative, especially for adolescents. Our results indicate that adolescent CU is associated with significantly lower 1/nT2* signal (consistent with lower tissue iron concentration) in subcortical regions involved in reinforcement, motivation, and addiction risk. Investigating neurobiology linked to adolescent CU like an estimate of subcortical tissue iron is crucial for identifying potential lasting effects of CU during development, including initiating other drug use [11], emerging mental health symptoms [78], and increased risk of developing CUD compared to adults [84].

## Supplementary information

Supplemental materials

## Data Availability

Participants did not consent to sharing their data.
