# Supplementary material for: The role of subcortical brain tissue iron as an indicator of dopamine neurophysiology in adolescent cannabis use
Source: Neuropsychopharmacology. 2026 Jun 2;51(11):2152–62. doi: 10.1038/s41386-026-02444-9 (PMC13597531; doi:10.1038/s41386-026-02444-9)
Supplement: Supplementary file 1 [file 41386_2026_2444_MOESM1_ESM.docx]

**Supplemental Material**

**Methods and Materials**

**MRI Processing**

Functional and anatomical data were preprocessed with the CONN Toolbox (version 22a) that runs in MATLAB (version R2023a; Natick, MA, The MathWorks, Inc.) using a flexible, standard preprocessing pipeline (1) with the following steps: realignment with correction of susceptibility distortion interactions, slice timing correction, outlier detection, segmentation into gray matter, white matter, and CSF (CONN Toolbox “direct segmentation” option), and MNI-space normalization. Functional data were realigned using the SPM realign & unwarp procedure (2); all scans were co-registered to a reference image (first scan of the first session) using a least squares approach and a 6-parameter (rigid body) transformation (3); resampled to correct for motion and magnetic susceptibility interactions; and temporally corrected via SPM slice-timing correction (4). Potential outlier scans were identified using ART (5) with a framewise displacement threshold of 0.5 mm (6, 7), and a reference BOLD image was computed for each subject by averaging all volumes, excluding outliers. Functional and anatomical data were normalized into standard MNI-152 space, segmented into gray matter, white matter, and cerebrospinal fluid (CSF) tissue classes, and resampled to 2 mm isotropic voxels following a direct normalization procedure (8) using the SPM unified segmentation and normalization algorithm (9, 10). Functional data were denoised using a standard pipeline(1) including regression of potential confounding effects (e.g., 5 CompCor components each for white matter and CSF), motion parameters (7, 11) and their first derivatives (12 factors), outliers, session effects with first derivatives, and linear effects (two factors each), bandpass filtering of the BOLD time series (12) between 0.008 Hz and 0.09 Hz, and despiking after denoising regression.

**Saliva Drug Test**

Participants completed a Narcocheck 5-panel saliva drug screen for tetrahydrocannabinol (THC), cocaine, opiates, amphetamines, and methamphetamines at the MRI visit. No adolescents tested positive for cannabis or other drugs during the MRI. Missing test results occurred for several reasons: saliva tests were not given to two pilot control participants scanned during the pandemic, though they denied substance use since their baseline visit; one control participant did not test because a test was unavailable at the MRI visit. Other missing results were due to invalid tests, often caused by equipment problems or insufficient saliva, evidenced by the middle test strip (THC) not showing results or outer strips failing to produce lines. For THC results, two adolescents in the cannabis group and six in the control group experienced equipment issues. Backup THC-only saliva tests, used when the 5-panel test failed, were given to four adolescents (one control, three cannabis), all testing negative. Therefore, of the total sample, 85.19% had negative THC results; 86.84% had negative cocaine and amphetamine results; 88.16% had negative opiate and methamphetamine results.

**Sensitivity and Post Hoc Follow-Up Analyses**

Sensitivity analyses tested whether significant effects remained the same when adjusting for slightly different scan acquisitions for n=5 participants by including a binary variable indicating pilot vs. main study; psychotropic medication (binary variable indicating whether medication was reported the day of the MRI); and nicotine use reported the day of the MRI (binary variable indicating yes or no). Post hoc follow-up analyses examined whether specific cannabis use (CU) factors were associated with 1/nT2* signal, including the formulation used (flower, concentrate) during the measurement period, recency of CU relative to 1/nT2* measurement, and lifetime cumulative exposure. Finally, we evaluated whether the same pattern of results was found when excluding participants with nicotine use during the TLFB measurement period. We followed the same linear mixed modeling analytic strategy for these analyses as for the primary analyses, and also included the same covariates, matching alcohol use and nicotine use to the same time frame as cannabis use when possible.

**Results**

**Sensitivity analyses**

The primary analyses were adjusted for factors that may influence the link between CU and 1/nT2*. When including the effect of study (pilot vs. main data collection), the same pattern of results remained. Separately, we adjusted for the influence of psychotropic medications reported on the MRI day by category (antipsychotic, antidepressant, stimulant, non-stimulant, and sleep medications; no one reported antianxiety or antiepileptic medications), and all aforementioned results remained significant. When excluding the four participants who reported nicotine use on the day of the MRI, patterns of results remained the same (Tables S8 and S9).

**Post hoc follow-up analyses**

We examined whether specific CU factors were associated with 1/nT2* signal, including the formulation used (flower, concentrate) during the measurement period, recency of CU relative to 1/nT2* measurement, and lifetime cumulative exposure. First, we assessed the proportion of days using concentrate and flower during the TLFB measurement period within the same model, finding that only concentrate days were significantly linked to lower 1/nT2* signal (Table S10). Post-hoc regressions showed that this relationship was specific to the VTA (Table S11).

Next, to ensure accuracy of the full TLFB recall period, sensitivity analyses for CU session data were tested in the 30 days prior to the 1/nT2* measurement, using the same covariates within the relevant time frame for nicotine and alcohol. A significant negative association emerged between cannabis sessions in the 30 days before the MRI and 1/nT2* signal (Table S12). Post-hoc regressions confirmed that recent CU sessions were significantly related to lower 1/nT2* signal in the left putamen (Table S13). We tested a mixed model for total lifetime episodes of concentrate use and smoked grams of cannabis, which was not significant (Table S14).

Finally, we tested whether the pattern of results remained the same when including only participants without nicotine use during the TLFB measurement period (44 controls and 12 cannabis-using teens remained). A similar pattern of results was found as the main analyses for daily concentrate hits, hours high, cannabis sessions, and CUD severity. Here however, there was also a significant relationship between daily smoked grams of flower and 1/nT2* (Tables S15-16). Post hoc regressions showed that more reported daily concentrate hits were associated with lower 1/nT2* in the left and right pallidum, left and right caudate, and VTA (Table S17); more daily smoked grams were linked to less 1/nT2* signal in the right nucleus accumbens and left pallidum (Table S18); more hours high was associated with less 1/nT2* signal in the left pallidum, left and right caudate, left putamen, and VTA (Table S19); more daily cannabis sessions were associated with lower 1/nT2* in the left pallidum, left and right caudate, left putamen, and VTA (Table S20); and higher cannabis use disorder (CUD) severity was related to lower 1/nT2* in the left and right caudate, left putamen, and VTA (Table S21).

**Table S1.** Participants’ (n) medication profile reported on the day of the MRI

| **Medication** | **N** |
| --- | --- |
| Sinupret, Loratadine; unspecified OTC allergy; hydrocortisone; montelukast; cetirizine hydrochloride | 9 |
| Hydroxyzine | 2 |
| Albuterol; beclomethasone dipropionate HFA inhaler | 2 |
| Melatonin | 5 |
| Iron, Zinc | 2 |
| Hydrochlorizide | 1 |
| Oral Contraceptive | 10 |
| Multi-vitamin, vitamin C, vitamin D , B-12; calcium | 10 |
| Fluoxetine; sertraline; duloxetine; escitalopram | 18 |
| Guanfacine, atamoxetine | 2 |
| Tylenol; Ibuprofen | 2 |
| Aripiprazole, olanzapine; aripiprazole | 3 |
| Testosterone; progestin and estrogen | 5 |
| Miralax | 1 |
| Methylphenidate | 3 |
| Dayquil | 1 |

Note. Participants reported more than one type of medication.

**Table S2:** Group descriptives and comparison of 1/nT2* by subcortical brain region

| **Variable** | **N** | **Control**  N = 47 | **Cannabis**  N = 34 | **Statistic** | **p-value** |
| --- | --- | --- | --- | --- | --- |
| **1/nT2* Region, Mean (SD)** |  |  |  |  |  |
| Ventral Tegmental Area | 81 | 0.98 (0.05) | 0.94 (0.04) | 3.74 | <0.001 |
| Left Accumbens | 81 | 0.84 (0.06) | 0.83 (0.04) | 1.51 | 0.136 |
| Right Accumbens | 81 | 0.86 (0.05) | 0.84 (0.04) | 2.63 | 0.010 |
| Left Caudate | 81 | 0.89 (0.03) | 0.87 (0.03) | 2.71 | 0.008 |
| Right Caudate | 81 | 0.90 (0.03) | 0.88 (0.03) | 2.92 | 0.005 |
| Left Putamen | 81 | 0.92 (0.03) | 0.90 (0.03) | 3.32 | 0.001 |
| Right Putamen | 81 | 0.94 (0.03) | 0.93 (0.03) | 1.56 | 0.123 |
| Left Thalamus | 81 | 0.92 (0.03) | 0.89 (0.02) | 4.38 | <0.001 |
| Right Thalamus | 81 | 0.93 (0.03) | 0.90 (0.02) | 4.13 | <0.001 |
| Left Pallidum | 81 | 1.24 (0.11) | 1.19 (0.08) | 2.01 | 0.048 |
| Right Pallidum | 81 | 1.25 (0.11) | 1.21 (0.08) | 1.82 | 0.072 |

Note. Statistics are Welch’s t-tests

**Table S3.** Post-hoc models specifying the link between daily concentrate hits and subcortical 1/nT2*

*****Left Accumbens*****

| **Variable** | **Beta** | **95% CI** | **p-value** | **q-value**^1^ |
| --- | --- | --- | --- | --- |
| Daily concentrate hits (log) | 0.00 | -0.02, 0.01 | 0.4 | 0.8 |
| Proportion of nicotine days (log) | -0.01 | -0.03, 0.02 | 0.7 | 0.8 |
| Proportion of standard drinks (log) | -0.02 | -0.05, 0.02 | 0.3 | 0.8 |
| Sex | 0.00 | -0.03, 0.02 | >0.9 | >0.9 |
| Age | 0.00 | -0.01, 0.02 | 0.4 | 0.8 |
| PLE | 0.00 | 0.00, 0.00 | 0.7 | 0.8 |
| ^1^False discovery rate correction for multiple testing | | | | |
| CI = Confidence Interval; PLE = psychotic-like experiences | | | | |

*****Right Accumbens*****

| **Variable** | **Beta** | **95% CI** | **p-value** | **q-value**^1^ |
| --- | --- | --- | --- | --- |
| Daily concentrate hits (log) | -0.01 | -0.01, 0.00 | 0.2 | 0.5 |
| Proportion of nicotine days (log) | -0.01 | -0.03, 0.01 | 0.3 | 0.5 |
| Proportion of standard drinks (log) | -0.01 | -0.04, 0.02 | 0.4 | 0.6 |
| Sex | 0.00 | -0.02, 0.02 | 0.8 | 0.8 |
| Age | 0.01 | 0.00, 0.02 | 0.3 | 0.5 |
| PLE | 0.00 | 0.00, 0.00 | 0.7 | 0.8 |
| ^1^False discovery rate correction for multiple testing | | | | |
| CI = Confidence Interval; PLE = psychotic-like experiences | | | | |

*****Left Pallidum*****

| **Variable** | **Beta** | **95% CI** | **p-value** | **q-value**^1^ |
| --- | --- | --- | --- | --- |
| Daily concentrate hits (log) | -0.03 | -0.05, -0.01 | 0.010 | 0.061 |
| Proportion of nicotine days (log) | 0.00 | -0.05, 0.04 | >0.9 | >0.9 |
| Proportion of standard drinks (log) | 0.07 | 0.00, 0.13 | 0.057 | 0.2 |
| Sex | -0.01 | -0.06, 0.04 | 0.6 | 0.8 |
| Age | 0.01 | -0.01, 0.04 | 0.3 | 0.5 |
| PLE | 0.00 | 0.00, 0.00 | 0.4 | 0.6 |
| ^1^False discovery rate correction for multiple testing | | | | |
| CI = Confidence Interval; PLE = psychotic-like experiences | | | | |

*****Right Pallidum*****

| **Variable** | **Beta** | **95% CI** | **p-value** | **q-value**^1^ |
| --- | --- | --- | --- | --- |
| Daily concentrate hits (log) | -0.02 | -0.04, 0.00 | 0.017 | 0.10 |
| Proportion of nicotine days (log) | -0.01 | -0.05, 0.03 | 0.7 | 0.8 |
| Proportion of standard drinks (log) | 0.07 | 0.00, 0.13 | 0.044 | 0.13 |
| Sex | 0.00 | -0.05, 0.05 | >0.9 | >0.9 |
| Age | 0.02 | 0.00, 0.04 | 0.12 | 0.2 |
| PLE | 0.00 | 0.00, 0.00 | 0.6 | 0.8 |
| ^1^False discovery rate correction for multiple testing | | | | |
| CI = Confidence Interval; PLE = psychotic-like experiences | | | | |

*****Left Caudate*****

| **Variable** | **Beta** | **95% CI** | **p-value** | **q-value**^1^ |
| --- | --- | --- | --- | --- |
| Daily concentrate hits (log) | -0.01 | -0.01, 0.00 | 0.074 | 0.4 |
| Proportion of nicotine days (log) | -0.01 | -0.02, 0.01 | 0.4 | >0.9 |
| Proportion of standard drinks (log) | 0.00 | -0.03, 0.02 | 0.8 | >0.9 |
| Sex | 0.00 | -0.02, 0.02 | >0.9 | >0.9 |
| Age | 0.00 | -0.01, 0.01 | 0.6 | >0.9 |
| PLE | 0.00 | 0.00, 0.00 | 0.6 | >0.9 |
| ^1^False discovery rate correction for multiple testing | | | | |
| CI = Confidence Interval; PLE = psychotic-like experiences | | | | |

*****Right Caudate*****

| **Variable** | **Beta** | **95% CI** | **p-value** | **q-value**^1^ |
| --- | --- | --- | --- | --- |
| Daily concentrate hits (log) | -0.01 | -0.01, 0.00 | 0.059 | 0.4 |
| Proportion of nicotine days (log) | -0.01 | -0.02, 0.01 | 0.5 | 0.9 |
| Proportion of standard drinks (log) | 0.00 | -0.03, 0.02 | 0.8 | 0.9 |
| Sex | 0.00 | -0.02, 0.02 | 0.8 | 0.9 |
| Age | 0.00 | -0.01, 0.01 | 0.9 | 0.9 |
| PLE | 0.00 | 0.00, 0.00 | 0.5 | 0.9 |
| ^1^False discovery rate correction for multiple testing | | | | |
| CI = Confidence Interval; PLE = psychotic-like experiences | | | | |

*****Left Putamen*****

| **Variable** | **Beta** | **95% CI** | **p-value** | **q-value**^1^ |
| --- | --- | --- | --- | --- |
| Daily concentrate hits (log) | -0.01 | -0.01, 0.00 | 0.051 | 0.3 |
| Proportion of nicotine days (log) | -0.01 | -0.02, 0.01 | 0.2 | 0.7 |
| Proportion of standard drinks (log) | 0.00 | -0.02, 0.02 | 0.9 | 0.9 |
| Sex | 0.00 | -0.02, 0.01 | 0.5 | 0.8 |
| Age | 0.00 | -0.01, 0.01 | 0.8 | 0.9 |
| PLE | 0.00 | 0.00, 0.00 | 0.5 | 0.8 |
| ^1^False discovery rate correction for multiple testing | | | | |
| CI = Confidence Interval; PLE = psychotic-like experiences | | | | |

*****Right Putamen*****

| **Variable** | **Beta** | **95% CI** | **p-value** | **q-value**^1^ |
| --- | --- | --- | --- | --- |
| Daily concentrate hits (log) | 0.00 | -0.01, 0.00 | 0.2 | 0.5 |
| Proportion of nicotine days (log) | -0.01 | -0.02, 0.01 | 0.3 | 0.5 |
| Proportion of standard drinks (log) | 0.01 | -0.01, 0.03 | 0.4 | 0.5 |
| Sex | 0.01 | -0.01, 0.02 | 0.3 | 0.5 |
| Age | 0.00 | 0.00, 0.01 | 0.3 | 0.5 |
| PLE | 0.00 | 0.00, 0.00 | 0.6 | 0.6 |
| ^1^False discovery rate correction for multiple testing | | | | |
| CI = Confidence Interval; PLE = psychotic-like experiences | | | | |

*****Left Thalamus*****

| **Variable** | **Beta** | **95% CI** | **p-value** | **q-value**^1^ |
| --- | --- | --- | --- | --- |
| Daily concentrate hits (log) | -0.01 | -0.01, 0.00 | 0.006 | 0.035 |
| Proportion of nicotine days (log) | 0.01 | 0.00, 0.02 | 0.2 | 0.3 |
| Proportion of standard drinks (log) | -0.01 | -0.03, 0.01 | 0.2 | 0.3 |
| Sex | -0.01 | -0.02, 0.01 | 0.4 | 0.4 |
| Age | 0.00 | 0.00, 0.01 | 0.3 | 0.4 |
| PLE | 0.00 | 0.00, 0.00 | 0.2 | 0.3 |
| ^1^False discovery rate correction for multiple testing | | | | |
| CI = Confidence Interval; PLE = psychotic-like experiences | | | | |

*****Right Thalamus*****

| **Variable** | **Beta** | **95% CI** | **p-value** | **q-value**^1^ |
| --- | --- | --- | --- | --- |
| Daily concentrate hits (log) | -0.01 | -0.01, 0.00 | 0.036 | 0.2 |
| Proportion of nicotine days (log) | 0.00 | -0.01, 0.01 | 0.7 | 0.7 |
| Proportion of standard drinks (log) | -0.01 | -0.03, 0.01 | 0.3 | 0.5 |
| Sex | 0.00 | -0.01, 0.02 | 0.5 | 0.6 |
| Age | 0.00 | 0.00, 0.01 | 0.4 | 0.5 |
| PLE | 0.00 | 0.00, 0.00 | 0.069 | 0.2 |
| ^1^False discovery rate correction for multiple testing | | | | |
| CI = Confidence Interval; PLE = psychotic-like experiences | | | | |

*****Ventral Tegmental Area*****

| **Variable** | **Beta** | **95% CI** | **p-value** | **q-value**^1^ |
| --- | --- | --- | --- | --- |
| Daily concentrate hits (log) | -0.01 | -0.02, 0.00 | 0.002 | 0.007 |
| Proportion of nicotine days (log) | 0.00 | -0.01, 0.02 | 0.7 | >0.9 |
| Proportion of standard drinks (log) | 0.00 | -0.03, 0.02 | 0.8 | >0.9 |
| Sex | -0.03 | -0.05, -0.01 | 0.002 | 0.007 |
| Age | 0.00 | -0.01, 0.01 | 0.4 | 0.8 |
| PLE | 0.00 | 0.00, 0.00 | >0.9 | >0.9 |
| ^1^False discovery rate correction for multiple testing | | | | |
| CI = Confidence Interval; PLE = psychotic-like experiences | | | | |

**Table S4.** Post-hoc models specifying the link between hours high and subcortical 1/nT2*

*****Left Accumbens*****

| **Variable** | **Beta** | **95% CI** | **p-value** | **q-value**^1^ |
| --- | --- | --- | --- | --- |
| Hours high ratio (log) | -0.01 | -0.03, 0.01 | 0.2 | 0.7 |
| Proportion of nicotine days (log) | 0.00 | -0.02, 0.03 | 0.8 | >0.9 |
| Proportion of standard drinks (log) | -0.02 | -0.05, 0.02 | 0.3 | 0.7 |
| Sex | 0.00 | -0.03, 0.02 | >0.9 | >0.9 |
| Age | 0.01 | -0.01, 0.02 | 0.3 | 0.7 |
| PLE | 0.00 | 0.00, 0.00 | 0.8 | >0.9 |
| ^1^False discovery rate correction for multiple testing | | | | |
| CI = Confidence Interval; PLE = psychotic-like experiences | | | | |

*****Right Accumbens*****

| **Variable** | **Beta** | **95% CI** | **p-value** | **q-value**^1^ |
| --- | --- | --- | --- | --- |
| Hours high ratio (log) | -0.01 | -0.02, 0.00 | 0.13 | 0.6 |
| Proportion of nicotine days (log) | -0.01 | -0.03, 0.01 | 0.4 | 0.6 |
| Proportion of standard drinks (log) | -0.01 | -0.04, 0.02 | 0.4 | 0.6 |
| Sex | 0.00 | -0.02, 0.02 | >0.9 | >0.9 |
| Age | 0.01 | 0.00, 0.02 | 0.2 | 0.6 |
| PLE | 0.00 | 0.00, 0.00 | 0.7 | 0.8 |
| ^1^False discovery rate correction for multiple testing | | | | |
| CI = Confidence Interval; PLE = psychotic-like experiences | | | | |

*****Left Pallidum*****

| **Variable** | **Beta** | **95% CI** | **p-value** | **q-value**^1^ |
| --- | --- | --- | --- | --- |
| Hours high ratio (log) | -0.03 | -0.07, 0.00 | 0.055 | 0.3 |
| Proportion of nicotine days (log) | 0.00 | -0.05, 0.05 | >0.9 | >0.9 |
| Proportion of standard drinks (log) | 0.06 | -0.01, 0.12 | 0.095 | 0.3 |
| Sex | -0.01 | -0.06, 0.04 | 0.7 | 0.8 |
| Age | 0.02 | -0.01, 0.04 | 0.2 | 0.4 |
| PLE | 0.00 | 0.00, 0.00 | 0.6 | 0.8 |
| ^1^False discovery rate correction for multiple testing | | | | |
| CI = Confidence Interval; PLE = psychotic-like experiences | | | | |

*****Right Pallidum*****

| **Variable** | **Beta** | **95% CI** | **p-value** | **q-value**^1^ |
| --- | --- | --- | --- | --- |
| Hours high ratio (log) | -0.02 | -0.05, 0.01 | 0.2 | 0.4 |
| Proportion of nicotine days (log) | -0.01 | -0.06, 0.03 | 0.5 | 0.8 |
| Proportion of standard drinks (log) | 0.05 | -0.01, 0.12 | 0.10 | 0.3 |
| Sex | 0.00 | -0.04, 0.05 | 0.9 | 0.9 |
| Age | 0.02 | 0.00, 0.04 | 0.071 | 0.3 |
| PLE | 0.00 | 0.00, 0.00 | 0.8 | 0.9 |
| ^1^False discovery rate correction for multiple testing | | | | |
| CI = Confidence Interval; PLE = psychotic-like experiences | | | | |

*****Left Caudate*****

| **Variable** | **Beta** | **95% CI** | **p-value** | **q-value**^1^ |
| --- | --- | --- | --- | --- |
| Hours high ratio (log) | -0.01 | -0.02, 0.00 | 0.2 | 0.8 |
| Proportion of nicotine days (log) | -0.01 | -0.02, 0.01 | 0.5 | 0.8 |
| Proportion of standard drinks (log) | 0.00 | -0.03, 0.02 | 0.7 | 0.8 |
| Sex | 0.00 | -0.02, 0.02 | >0.9 | >0.9 |
| Age | 0.00 | -0.01, 0.01 | 0.5 | 0.8 |
| PLE | 0.00 | 0.00, 0.00 | 0.7 | 0.8 |
| ^1^False discovery rate correction for multiple testing | | | | |
| CI = Confidence Interval; PLE = psychotic-like experiences | | | | |

*****Right Caudate*****

| **Variable** | **Beta** | **95% CI** | **p-value** | **q-value**^1^ |
| --- | --- | --- | --- | --- |
| Hours high ratio (log) | -0.01 | -0.02, 0.01 | 0.4 | 0.9 |
| Proportion of nicotine days (log) | -0.01 | -0.03, 0.01 | 0.3 | 0.9 |
| Proportion of standard drinks (log) | -0.01 | -0.03, 0.02 | 0.6 | 0.9 |
| Sex | 0.00 | -0.02, 0.02 | 0.9 | 0.9 |
| Age | 0.00 | -0.01, 0.01 | 0.8 | 0.9 |
| PLE | 0.00 | 0.00, 0.00 | 0.7 | 0.9 |
| ^1^False discovery rate correction for multiple testing | | | | |
| CI = Confidence Interval; PLE = psychotic-like experiences | | | | |

*****Left Putamen*****

| **Variable** | **Beta** | **95% CI** | **p-value** | **q-value**^1^ |
| --- | --- | --- | --- | --- |
| Hours high ratio (log) | -0.01 | -0.02, 0.00 | 0.051 | 0.3 |
| Proportion of nicotine days (log) | -0.01 | -0.02, 0.01 | 0.4 | 0.7 |
| Proportion of standard drinks (log) | 0.00 | -0.02, 0.02 | >0.9 | >0.9 |
| Sex | -0.01 | -0.02, 0.01 | 0.4 | 0.7 |
| Age | 0.00 | -0.01, 0.01 | 0.6 | 0.7 |
| PLE | 0.00 | 0.00, 0.00 | 0.6 | 0.7 |
| ^1^False discovery rate correction for multiple testing | | | | |
| CI = Confidence Interval; PLE = psychotic-like experiences | | | | |

*****Right Putamen*****

| **Variable** | **Beta** | **95% CI** | **p-value** | **q-value**^1^ |
| --- | --- | --- | --- | --- |
| Hours high ratio (log) | 0.00 | -0.02, 0.01 | 0.4 | 0.6 |
| Proportion of nicotine days (log) | -0.01 | -0.02, 0.01 | 0.4 | 0.6 |
| Proportion of standard drinks (log) | 0.01 | -0.02, 0.03 | 0.5 | 0.6 |
| Sex | 0.01 | -0.01, 0.02 | 0.3 | 0.6 |
| Age | 0.01 | 0.00, 0.01 | 0.2 | 0.6 |
| PLE | 0.00 | 0.00, 0.00 | 0.6 | 0.6 |
| ^1^False discovery rate correction for multiple testing | | | | |
| CI = Confidence Interval; PLE = psychotic-like experiences | | | | |

*****Left Thalamus*****

| **Variable** | **Beta** | **95% CI** | **p-value** | **q-value**^1^ |
| --- | --- | --- | --- | --- |
| Hours high ratio (log) | -0.01 | -0.02, 0.00 | 0.060 | 0.2 |
| Proportion of nicotine days (log) | 0.01 | -0.01, 0.02 | 0.3 | 0.3 |
| Proportion of standard drinks (log) | -0.01 | -0.03, 0.00 | 0.10 | 0.2 |
| Sex | -0.01 | -0.02, 0.01 | 0.4 | 0.4 |
| Age | 0.00 | 0.00, 0.01 | 0.2 | 0.3 |
| PLE | 0.00 | 0.00, 0.00 | 0.11 | 0.2 |
| ^1^False discovery rate correction for multiple testing | | | | |
| CI = Confidence Interval; PLE = psychotic-like experiences | | | | |

*****Right Thalamus*****

| **Variable** | **Beta** | **95% CI** | **p-value** | **q-value**^1^ |
| --- | --- | --- | --- | --- |
| Hours high ratio (log) | -0.01 | -0.02, 0.00 | 0.2 | 0.3 |
| Proportion of nicotine days (log) | 0.00 | -0.01, 0.01 | 0.9 | 0.9 |
| Proportion of standard drinks (log) | -0.01 | -0.03, 0.01 | 0.2 | 0.3 |
| Sex | 0.00 | -0.01, 0.02 | 0.6 | 0.7 |
| Age | 0.00 | 0.00, 0.01 | 0.15 | 0.3 |
| PLE | 0.00 | 0.00, 0.00 | 0.080 | 0.3 |
| ^1^False discovery rate correction for multiple testing | | | | |
| CI = Confidence Interval; PLE = psychotic-like experiences | | | | |

*****Ventral Tegmental Area*****

| **Variable** | **Beta** | **95% CI** | **p-value** | **q-value**^1^ |
| --- | --- | --- | --- | --- |
| Hours high ratio (log) | -0.01 | -0.02, 0.01 | 0.2 | 0.4 |
| Proportion of nicotine days (log) | 0.00 | -0.02, 0.02 | >0.9 | >0.9 |
| Proportion of standard drinks (log) | -0.01 | -0.04, 0.02 | 0.5 | 0.7 |
| Sex | -0.03 | -0.05, -0.01 | 0.004 | 0.026 |
| Age | 0.01 | 0.00, 0.02 | 0.2 | 0.4 |
| PLE | 0.00 | 0.00, 0.00 | 0.7 | 0.9 |
| ^1^False discovery rate correction for multiple testing | | | | |
| CI = Confidence Interval; PLE = psychotic-like experiences | | | | |

**Table S5.** Post-hoc models specifying the link between cannabis frequency and subcortical 1/nT2*

*****Left Accumbens*****

| **Variable** | **Beta** | **95% CI** | **p-value** | **q-value**^1^ |
| --- | --- | --- | --- | --- |
| Cannabis sessions ratio (log) | -0.01 | -0.03, 0.01 | 0.2 | 0.7 |
| Proportion of nicotine days (log) | 0.00 | -0.02, 0.03 | 0.8 | >0.9 |
| Proportion of standard drinks (log) | -0.02 | -0.05, 0.02 | 0.3 | 0.7 |
| Sex | 0.00 | -0.03, 0.02 | >0.9 | >0.9 |
| Age | 0.01 | -0.01, 0.02 | 0.3 | 0.7 |
| PLE | 0.00 | 0.00, 0.00 | 0.8 | >0.9 |
| ^1^False discovery rate correction for multiple testing | | | | |
| CI = Confidence Interval; PLE = psychotic-like experiences | | | | |

*****Right Accumbens*****

| **Variable** | **Beta** | **95% CI** | **p-value** | **q-value**^1^ |
| --- | --- | --- | --- | --- |
| Cannabis sessions ratio (log) | -0.01 | -0.03, 0.01 | 0.3 | 0.5 |
| Proportion of nicotine days (log) | -0.01 | -0.03, 0.01 | 0.3 | 0.5 |
| Proportion of standard drinks (log) | -0.01 | -0.04, 0.02 | 0.4 | 0.5 |
| Sex | 0.00 | -0.02, 0.02 | 0.9 | 0.9 |
| Age | 0.01 | 0.00, 0.02 | 0.2 | 0.5 |
| PLE | 0.00 | 0.00, 0.00 | 0.6 | 0.7 |
| ^1^False discovery rate correction for multiple testing | | | | |
| CI = Confidence Interval; PLE = psychotic-like experiences | | | | |

*****Left Pallidum*****

| **Variable** | **Beta** | **95% CI** | **p-value** | **q-value**^1^ |
| --- | --- | --- | --- | --- |
| Cannabis sessions ratio (log) | -0.05 | -0.08, -0.01 | 0.018 | 0.11 |
| Proportion of nicotine days (log) | 0.01 | -0.04, 0.06 | 0.8 | 0.8 |
| Proportion of standard drinks (log) | 0.06 | 0.00, 0.13 | 0.065 | 0.2 |
| Sex | -0.01 | -0.06, 0.04 | 0.6 | 0.7 |
| Age | 0.02 | -0.01, 0.04 | 0.2 | 0.4 |
| PLE | 0.00 | 0.00, 0.00 | 0.6 | 0.7 |
| ^1^False discovery rate correction for multiple testing | | | | |
| CI = Confidence Interval; PLE = psychotic-like experiences | | | | |

*****Right Pallidum*****

| **Variable** | **Beta** | **95% CI** | **p-value** | **q-value**^1^ |
| --- | --- | --- | --- | --- |
| Cannabis sessions ratio (log) | -0.03 | -0.06, 0.01 | 0.13 | 0.3 |
| Proportion of nicotine days (log) | -0.01 | -0.06, 0.04 | 0.7 | >0.9 |
| Proportion of standard drinks (log) | 0.06 | -0.01, 0.12 | 0.084 | 0.3 |
| Sex | 0.00 | -0.05, 0.05 | >0.9 | >0.9 |
| Age | 0.02 | 0.00, 0.04 | 0.067 | 0.3 |
| PLE | 0.00 | 0.00, 0.00 | 0.8 | >0.9 |
| ^1^False discovery rate correction for multiple testing | | | | |
| CI = Confidence Interval; PLE = psychotic-like experiences | | | | |

*****Left Caudate*****

| **Variable** | **Beta** | **95% CI** | **p-value** | **q-value**^1^ |
| --- | --- | --- | --- | --- |
| Cannabis sessions ratio (log) | -0.01 | -0.02, 0.00 | 0.2 | 0.9 |
| Proportion of nicotine days (log) | -0.01 | -0.02, 0.01 | 0.5 | 0.9 |
| Proportion of standard drinks (log) | 0.00 | -0.03, 0.02 | 0.7 | 0.9 |
| Sex | 0.00 | -0.02, 0.02 | 0.9 | 0.9 |
| Age | 0.00 | 0.00, 0.01 | 0.5 | 0.9 |
| PLE | 0.00 | 0.00, 0.00 | 0.7 | 0.9 |
| ^1^False discovery rate correction for multiple testing | | | | |
| CI = Confidence Interval; PLE = psychotic-like experiences | | | | |

*****Right Caudate*****

| **Variable** | **Beta** | **95% CI** | **p-value** | **q-value**^1^ |
| --- | --- | --- | --- | --- |
| Cannabis sessions ratio (log) | -0.01 | -0.02, 0.01 | 0.4 | 0.9 |
| Proportion of nicotine days (log) | -0.01 | -0.03, 0.01 | 0.3 | 0.9 |
| Proportion of standard drinks (log) | -0.01 | -0.03, 0.02 | 0.6 | 0.9 |
| Sex | 0.00 | -0.02, 0.02 | 0.9 | 0.9 |
| Age | 0.00 | -0.01, 0.01 | 0.8 | 0.9 |
| PLE | 0.00 | 0.00, 0.00 | 0.8 | 0.9 |
| ^1^False discovery rate correction for multiple testing | | | | |
| CI = Confidence Interval; PLE = psychotic-like experiences | | | | |

*****Left Putamen*****

| **Variable** | **Beta** | **95% CI** | **p-value** | **q-value**^1^ |
| --- | --- | --- | --- | --- |
| Cannabis sessions ratio (log) | -0.01 | -0.02, 0.00 | 0.041 | 0.2 |
| Proportion of nicotine days (log) | -0.01 | -0.02, 0.01 | 0.5 | 0.7 |
| Proportion of standard drinks (log) | 0.00 | -0.02, 0.02 | 0.9 | 0.9 |
| Sex | -0.01 | -0.02, 0.01 | 0.4 | 0.7 |
| Age | 0.00 | -0.01, 0.01 | 0.6 | 0.7 |
| PLE | 0.00 | 0.00, 0.00 | 0.6 | 0.7 |
| ^1^False discovery rate correction for multiple testing | | | | |
| CI = Confidence Interval; PLE = psychotic-like experiences | | | | |

*****Right Putamen*****

| **Variable** | **Beta** | **95% CI** | **p-value** | **q-value**^1^ |
| --- | --- | --- | --- | --- |
| Cannabis sessions ratio (log) | -0.01 | -0.02, 0.01 | 0.4 | 0.6 |
| Proportion of nicotine days (log) | -0.01 | -0.02, 0.01 | 0.5 | 0.6 |
| Proportion of standard drinks (log) | 0.01 | -0.01, 0.03 | 0.5 | 0.6 |
| Sex | 0.01 | -0.01, 0.02 | 0.4 | 0.6 |
| Age | 0.01 | 0.00, 0.01 | 0.2 | 0.6 |
| PLE | 0.00 | 0.00, 0.00 | 0.6 | 0.6 |
| ^1^False discovery rate correction for multiple testing | | | | |
| CI = Confidence Interval; PLE = psychotic-like experiences | | | | |

*****Left Thalamus*****

| **Variable** | **Beta** | **95% CI** | **p-value** | **q-value**^1^ |
| --- | --- | --- | --- | --- |
| Cannabis sessions ratio (log) | -0.01 | -0.02, 0.00 | 0.10 | 0.2 |
| Proportion of nicotine days (log) | 0.01 | -0.01, 0.02 | 0.3 | 0.4 |
| Proportion of standard drinks (log) | -0.01 | -0.03, 0.00 | 0.10 | 0.2 |
| Sex | -0.01 | -0.02, 0.01 | 0.4 | 0.4 |
| Age | 0.00 | 0.00, 0.01 | 0.2 | 0.3 |
| PLE | 0.00 | 0.00, 0.00 | 0.084 | 0.2 |
| ^1^False discovery rate correction for multiple testing | | | | |
| CI = Confidence Interval; PLE = psychotic-like experiences | | | | |

*****Right Thalamus*****

| **Variable** | **Beta** | **95% CI** | **p-value** | **q-value**^1^ |
| --- | --- | --- | --- | --- |
| Cannabis sessions ratio (log) | -0.01 | -0.02, 0.01 | 0.3 | 0.5 |
| Proportion of nicotine days (log) | 0.00 | -0.02, 0.01 | 0.8 | 0.8 |
| Proportion of standard drinks (log) | -0.01 | -0.03, 0.01 | 0.2 | 0.4 |
| Sex | 0.00 | -0.01, 0.02 | 0.6 | 0.7 |
| Age | 0.00 | 0.00, 0.01 | 0.2 | 0.4 |
| PLE | 0.00 | 0.00, 0.00 | 0.062 | 0.4 |
| ^1^False discovery rate correction for multiple testing | | | | |
| CI = Confidence Interval; PLE = psychotic-like experiences | | | | |

*****Ventral Tegmental Area*****

| **Variable** | **Beta** | **95% CI** | **p-value** | **q-value**^1^ |
| --- | --- | --- | --- | --- |
| Cannabis sessions ratio (log) | -0.01 | -0.03, 0.01 | 0.2 | 0.4 |
| Proportion of nicotine days (log) | 0.00 | -0.02, 0.02 | >0.9 | >0.9 |
| Proportion of standard drinks (log) | -0.01 | -0.04, 0.02 | 0.5 | 0.7 |
| Sex | -0.03 | -0.05, -0.01 | 0.004 | 0.024 |
| Age | 0.01 | 0.00, 0.02 | 0.2 | 0.4 |
| PLE | 0.00 | 0.00, 0.00 | 0.7 | 0.8 |
| ^1^False discovery rate correction for multiple testing | | | | |
| CI = Confidence Interval; PLE = psychotic-like experiences | | | | |

**Table S6.** Post-hoc models specifying the link between CUD severity and subcortical 1/nT2*

*****Left Accumbens*****

| **Variable** | **Beta** | **95% CI** | **p-value** | **q-value**^1^ |
| --- | --- | --- | --- | --- |
| CUD severity | -0.01 | -0.02, 0.00 | 0.2 | 0.5 |
| Proportion of nicotine days (log) | 0.00 | -0.02, 0.02 | >0.9 | >0.9 |
| Proportion of standard drinks (log) | -0.02 | -0.05, 0.01 | 0.2 | 0.5 |
| Sex | 0.00 | -0.02, 0.02 | >0.9 | >0.9 |
| Age | 0.01 | 0.00, 0.02 | 0.2 | 0.5 |
| PLE | 0.00 | 0.00, 0.00 | 0.8 | >0.9 |
| ^1^False discovery rate correction for multiple testing | | | | |
| CI = Confidence Interval; PLE = psychotic-like experiences; CUD = cannabis use disorder | | | | |

*****Right Accumbens*****

| **Variable** | **Beta** | **95% CI** | **p-value** | **q-value**^1^ |
| --- | --- | --- | --- | --- |
| CUD severity | -0.01 | -0.02, 0.00 | 0.2 | 0.4 |
| Proportion of nicotine days (log) | -0.01 | -0.03, 0.01 | 0.2 | 0.4 |
| Proportion of standard drinks (log) | -0.02 | -0.04, 0.01 | 0.3 | 0.4 |
| Sex | 0.00 | -0.02, 0.02 | 0.9 | 0.9 |
| Age | 0.01 | 0.00, 0.02 | 0.2 | 0.4 |
| PLE | 0.00 | 0.00, 0.00 | 0.6 | 0.7 |
| ^1^False discovery rate correction for multiple testing | | | | |
| CI = Confidence Interval; PLE = psychotic-like experiences; CUD = cannabis use disorder | | | | |

*****Left Pallidum*****

| **Variable** | **Beta** | **95% CI** | **p-value** | **q-value**^1^ |
| --- | --- | --- | --- | --- |
| CUD severity | -0.03 | -0.06, -0.01 | 0.012 | 0.070 |
| Proportion of nicotine days (log) | 0.00 | -0.05, 0.05 | >0.9 | >0.9 |
| Proportion of standard drinks (log) | 0.05 | -0.01, 0.12 | 0.12 | 0.3 |
| Sex | -0.01 | -0.06, 0.04 | 0.6 | 0.7 |
| Age | 0.02 | -0.01, 0.04 | 0.14 | 0.3 |
| PLE | 0.00 | 0.00, 0.00 | 0.6 | 0.7 |
| ^1^False discovery rate correction for multiple testing | | | | |
| CI = Confidence Interval; PLE = psychotic-like experiences; CUD = cannabis use disorder | | | | |

*****Right Pallidum*****

| **Variable** | **Beta** | **95% CI** | **p-value** | **q-value**^1^ |
| --- | --- | --- | --- | --- |
| CUD severity | -0.03 | -0.05, 0.00 | 0.028 | 0.14 |
| Proportion of nicotine days (log) | -0.01 | -0.05, 0.04 | 0.8 | >0.9 |
| Proportion of standard drinks (log) | 0.05 | -0.01, 0.11 | 0.10 | 0.2 |
| Sex | 0.00 | -0.05, 0.05 | >0.9 | >0.9 |
| Age | 0.02 | 0.00, 0.04 | 0.045 | 0.14 |
| PLE | 0.00 | 0.00, 0.00 | 0.7 | >0.9 |
| ^1^False discovery rate correction for multiple testing | | | | |
| CI = Confidence Interval; PLE = psychotic-like experiences; CUD = cannabis use disorder | | | | |

*****Left Caudate*****

| **Variable** | **Beta** | **95% CI** | **p-value** | **q-value**^1^ |
| --- | --- | --- | --- | --- |
| CUD severity | -0.01 | -0.02, 0.00 | 0.073 | 0.4 |
| Proportion of nicotine days (log) | -0.01 | -0.02, 0.01 | 0.5 | 0.8 |
| Proportion of standard drinks (log) | -0.01 | -0.03, 0.02 | 0.6 | 0.8 |
| Sex | 0.00 | -0.02, 0.02 | 0.9 | 0.9 |
| Age | 0.00 | 0.00, 0.01 | 0.4 | 0.8 |
| PLE | 0.00 | 0.00, 0.00 | 0.6 | 0.8 |
| ^1^False discovery rate correction for multiple testing | | | | |
| CI = Confidence Interval; PLE = psychotic-like experiences; CUD = cannabis use disorder | | | | |

*****Right Caudate*****

| **Variable** | **Beta** | **95% CI** | **p-value** | **q-value**^1^ |
| --- | --- | --- | --- | --- |
| CUD severity | -0.01 | -0.02, 0.00 | 0.14 | 0.8 |
| Proportion of nicotine days (log) | -0.01 | -0.02, 0.01 | 0.4 | 0.8 |
| Proportion of standard drinks (log) | -0.01 | -0.03, 0.02 | 0.6 | 0.8 |
| Sex | 0.00 | -0.02, 0.02 | >0.9 | >0.9 |
| Age | 0.00 | -0.01, 0.01 | 0.7 | 0.8 |
| PLE | 0.00 | 0.00, 0.00 | 0.7 | 0.8 |
| ^1^False discovery rate correction for multiple testing | | | | |
| CI = Confidence Interval; PLE = psychotic-like experiences; CUD = cannabis use disorder | | | | |

*****Left Putamen*****

| **Variable** | **Beta** | **95% CI** | **p-value** | **q-value**^1^ |
| --- | --- | --- | --- | --- |
| CUD severity | -0.01 | -0.02, 0.00 | 0.087 | 0.5 |
| Proportion of nicotine days (log) | -0.01 | -0.02, 0.01 | 0.2 | 0.6 |
| Proportion of standard drinks (log) | 0.00 | -0.02, 0.02 | 0.8 | 0.8 |
| Sex | -0.01 | -0.02, 0.01 | 0.5 | 0.8 |
| Age | 0.00 | -0.01, 0.01 | 0.6 | 0.8 |
| PLE | 0.00 | 0.00, 0.00 | 0.7 | 0.8 |
| ^1^False discovery rate correction for multiple testing | | | | |
| CI = Confidence Interval; PLE = psychotic-like experiences; CUD = cannabis use disorder | | | | |

*****Right Putamen*****

| **Variable** | **Beta** | **95% CI** | **p-value** | **q-value**^1^ |
| --- | --- | --- | --- | --- |
| CUD severity | 0.00 | -0.01, 0.01 | 0.7 | 0.7 |
| Proportion of nicotine days (log) | -0.01 | -0.02, 0.01 | 0.2 | 0.6 |
| Proportion of standard drinks (log) | 0.00 | -0.02, 0.03 | 0.7 | 0.7 |
| Sex | 0.01 | -0.01, 0.02 | 0.3 | 0.6 |
| Age | 0.01 | 0.00, 0.01 | 0.2 | 0.6 |
| PLE | 0.00 | 0.00, 0.00 | 0.7 | 0.7 |
| ^1^False discovery rate correction for multiple testing | | | | |
| CI = Confidence Interval; PLE = psychotic-like experiences; CUD = cannabis use disorder | | | | |

*****Left Thalamus*****

| **Variable** | **Beta** | **95% CI** | **p-value** | **q-value**^1^ |
| --- | --- | --- | --- | --- |
| CUD severity | 0.00 | -0.01, 0.00 | 0.2 | 0.3 |
| Proportion of nicotine days (log) | 0.00 | -0.01, 0.02 | 0.6 | 0.6 |
| Proportion of standard drinks (log) | -0.02 | -0.03, 0.00 | 0.049 | 0.2 |
| Sex | 0.00 | -0.02, 0.01 | 0.5 | 0.5 |
| Age | 0.00 | 0.00, 0.01 | 0.2 | 0.3 |
| PLE | 0.00 | 0.00, 0.00 | 0.074 | 0.2 |
| ^1^False discovery rate correction for multiple testing | | | | |
| CI = Confidence Interval; PLE = psychotic-like experiences; CUD = cannabis use disorder | | | | |

*****Right Thalamus*****

| **Variable** | **Beta** | **95% CI** | **p-value** | **q-value**^1^ |
| --- | --- | --- | --- | --- |
| CUD severity | 0.00 | -0.01, 0.01 | 0.8 | 0.8 |
| Proportion of nicotine days (log) | -0.01 | -0.02, 0.01 | 0.4 | 0.5 |
| Proportion of standard drinks (log) | -0.01 | -0.03, 0.00 | 0.12 | 0.3 |
| Sex | 0.01 | -0.01, 0.02 | 0.5 | 0.5 |
| Age | 0.00 | 0.00, 0.01 | 0.2 | 0.3 |
| PLE | 0.00 | 0.00, 0.00 | 0.048 | 0.3 |
| ^1^False discovery rate correction for multiple testing | | | | |
| CI = Confidence Interval; PLE = psychotic-like experiences; CUD = cannabis use disorder | | | | |

*****Ventral Tegmental Area*****

| **Variable** | **Beta** | **95% CI** | **p-value** | **q-value**^1^ |
| --- | --- | --- | --- | --- |
| CUD severity | -0.02 | -0.03, 0.00 | 0.006 | 0.018 |
| Proportion of nicotine days (log) | 0.01 | -0.01, 0.02 | 0.5 | 0.7 |
| Proportion of standard drinks (log) | -0.01 | -0.04, 0.02 | 0.4 | 0.6 |
| Sex | -0.03 | -0.05, -0.01 | 0.001 | 0.009 |
| Age | 0.01 | 0.00, 0.02 | 0.13 | 0.3 |
| PLE | 0.00 | 0.00, 0.00 | 0.9 | 0.9 |
| ^1^False discovery rate correction for multiple testing | | | | |
| CI = Confidence Interval; PLE = psychotic-like experiences; CUD = cannabis use disorder | | | | |

**Table S7a.** The association between cannabis quantity (models 1 and 2) and subjective intoxication (model 3) with subcortical 1/nT2* signal intensity, including covariates

|  | **Model 1** (n=76) | | | **Model 2** (n=78) | | | **Model 3** (n=78) | | |
| --- | --- | --- | --- | --- | --- | --- | --- | --- | --- |
| *Predictors* | *Estimates* | *CI (95%)* | *p* | *Estimates* | *CI (95%)* | *p* | *Estimates* | *CI (95%)* | *p* |
| Intercept | 0.87 | 0.73 – 1.01 | **<0.001** | 0.83 | 0.69 – 0.98 | **<0.001** | 0.84 | 0.70 – 0.98 | **<0.001** |
| Daily concentrate hits (log) | -0.01 | -0.02 – -0.00 | **<0.001** |  |  |  |  |  |  |
| Proportion of nicotine days (log) | -0.00 | -0.02 – 0.01 | 0.491 | -0.01 | -0.02 – 0.00 | 0.075 | -0.00 | -0.02 – 0.01 | 0.552 |
| Proportion of standard drinks (log) | 0.01 | -0.01 – 0.03 | 0.405 | -0.00 | -0.02 – 0.02 | 0.982 | 0.00 | -0.01 – 0.02 | 0.691 |
| Sex | -0.00 | -0.02 – 0.01 | 0.599 | -0.00 | -0.01 – 0.01 | 0.880 | -0.00 | -0.02 – 0.01 | 0.610 |
| Age | 0.01 | -0.00 – 0.01 | 0.093 | 0.01 | -0.00 – 0.01 | 0.062 | 0.01 | 0.00 – 0.01 | **0.038** |
| PLE | 0.00 | -0.00 – 0.00 | 0.760 | -0.00 | -0.00 – 0.00 | 0.961 | 0.00 | -0.00 – 0.00 | 0.996 |
| Daily cannabis grams (log) |  |  |  | -0.01 | -0.02 – 0.00 | 0.094 |  |  |  |
| Daily hours high ratio (log) |  |  |  |  |  |  | -0.01 | -0.02 – -0.00 | **0.016** |

Note. Each model was run separately. This table shows the effects in Table 2, with covariates depicted. CI = Confidence Interval; PLE = psychotic-like experiences

**Table S7b.** The association between cannabis frequency (model 4) and cannabis use disorder severity (model 5) with subcortical 1/nT2* signal intensity, including covariates

|  | **Model 4** (n=78) | | | **Model 5** (n=78) | | |
| --- | --- | --- | --- | --- | --- | --- |
| *Predictors* | *Estimates* | *CI (95%)* | *p* | *Estimates* | *CI (95%)* | *p* |
| Intercept | 0.84 | 0.70 – 0.99 | **<0.001** | 0.86 | 0.72 – 1.00 | **<0.001** |
| Cannabis sessions ratio (log) | -0.01 | -0.02 – -0.00 | **0.010** |  |  |  |
| Proportion of nicotine days (log) | -0.00 | -0.02 – 0.01 | 0.701 | -0.00 | -0.02 – 0.01 | 0.496 |
| Proportion of standard drinks (log) | 0.00 | -0.01 – 0.02 | 0.616 | 0.00 | -0.02 – 0.02 | 0.894 |
| Sex | -0.00 | -0.02 – 0.01 | 0.576 | -0.00 | -0.02 – 0.01 | 0.569 |
| Age | 0.01 | 0.00 – 0.01 | **0.036** | 0.01 | 0.00 – 0.01 | **0.023** |
| PLE | -0.00 | -0.00 – 0.00 | 0.946 | -0.00 | -0.00 – 0.00 | 0.989 |
| CUD severity |  |  |  | -0.01 | -0.02 – -0.00 | **0.003** |

Note. Each model was run separately. This table shows the effects in Table 2, with covariates depicted. CI = Confidence Interval; CUD = cannabis use disorder; PLE = psychotic-like experiences

**Table S8.** The association between cannabis quantity (models 1 and 2) and subjective intoxication (model 3) with subcortical 1/nT2* signal intensity when excluding participants (n=4) with nicotine use at the MRI

|  | **Model 1** (n = 73) | | | **Model 2** (n = 74) | | | **Model 3** (n = 74) | | |
| --- | --- | --- | --- | --- | --- | --- | --- | --- | --- |
| *Predictors* | *Estimates* | *CI (95%)* | *p* | *Estimates* | *CI (95%)* | *p* | *Estimates* | *CI (95%)* | *p* |
| Intercept | 0.90 | 0.75 – 1.05 | **<0.001** | 0.87 | 0.71 – 1.02 | **<0.001** | 0.88 | 0.73 – 1.03 | **<0.001** |
| Daily concentrate hits (log) | -0.01 | -0.02 – <-0.01 | **<0.001** |  |  |  |  |  |  |
| Proportion of nicotine days (log) | -0.00 | -0.01 – 0.01 | 0.939 | -0.01 | -0.02 – 0.01 | 0.261 | -0.00 | -0.02 – 0.01 | 0.849 |
| Proportion of standard drinks (log) | 0.01 | -0.01 – 0.03 | 0.243 | 0.00 | -0.02 – 0.02 | 0.701 | 0.01 | -0.01 – 0.03 | 0.448 |
| Sex | -0.00 | -0.02 – 0.01 | 0.562 | -0.00 | -0.02 – 0.01 | 0.779 | -0.00 | -0.02 – 0.01 | 0.542 |
| Age | 0.00 | -0.00 – 0.01 | 0.206 | 0.01 | -0.00 – 0.01 | 0.154 | 0.01 | -0.00 – 0.01 | 0.123 |
| PLE | 0.00 | -0.00 – 0.00 | 0.750 | -0.00 | -0.00 – 0.00 | 0.890 | -0.00 | -0.00 – 0.00 | 0.960 |
| Daily cannabis grams (log) |  |  |  | -0.01 | -0.02 – 0.00 | 0.103 |  |  |  |
| Daily hours high ratio (log) |  |  |  |  |  |  | -0.01 | -0.02 – < -0.01 | **0.015** |

Note. CI = Confidence Interval; PLE = psychotic-like experiences

**Table S9.** The association between cannabis sessions (model 1) and cannabis use disorder severity (model 2) with subcortical 1/nT2* signal intensity when excluding participants (n=4) with nicotine use at the MRI

|  | **Model 1** (n = 74) | | | **Model 2** (n = 74) | | |
| --- | --- | --- | --- | --- | --- | --- |
| *Predictors* | *Estimates* | *CI (95%)* | *p* | *Estimates* | *CI (95%)* | *p* |
| Intercept | 0.88 | 0.73 – 1.03 | **<0.001** | 0.89 | 0.74 – 1.04 | **<0.001** |
| Cannabis sessions ratio (log) | -0.01 | -0.02 – < -0.01 | **0.011** |  |  |  |
| Proportion of nicotine days (log) | < -0.01 | -0.02 – 0.02 | 0.967 | < -0.01 | -0.02 – 0.01 | 0.653 |
| Proportion of standard drinks (log) | 0.01 | -0.01 – 0.03 | 0.406 | 0.00 | -0.01 – 0.02 | 0.666 |
| Sex | < -0.01 | -0.02 – 0.01 | 0.527 | < -0.01 | -0.02 – 0.01 | 0.553 |
| Age | 0.01 | < -0.01 – 0.01 | 0.117 | 0.01 | < -0.01 – 0.01 | 0.077 |
| PLE | < -0.01 | < -0.01 – 0.00 | 0.899 | < -0.01 | < -0.01 – 0.00 | 0.883 |
| CUD severity |  |  |  | -0.01 | -0.02 – < -0.01 | **0.004** |

Note. CI = Confidence Interval; CUD = cannabis use disorder; PLE = psychotic-like experiences

**Table S10.** The association between proportion of concentrate and flower use days over the measurement period with subcortical 1/nT2* signal intensity within the same model

|  | (n = 78) | | |
| --- | --- | --- | --- |
| *Predictors* | *Estimates* | *CI (95%)* | *p* |
| Intercept | 0.81 | 0.66 – 0.95 | **<0.001** |
| Proportion of concentrate days ratio (log) | -0.02 | -0.03 – < -0.01 | **0.017** |
| Proportion of flower days ratio (log) | -0.01 | -0.02 – 0.01 | 0.361 |
| Proportion of nicotine days (log) | < -0.01 | -0.02 – 0.01 | 0.635 |
| Proportion of standard drinks (log) | 0.00 | -0.02 – 0.02 | 0.892 |
| Sex | < -0.01 | -0.02 – 0.01 | 0.525 |
| Age | 0.01 | 0.00 – 0.01 | **0.030** |
| PLE | 0.00 | < -0.01 – 0.00 | 0.877 |

Note. CI = Confidence Interval; PLE = psychotic-like experiences

**Table S11.** Post-hoc models specifying the link between cannabis formulation and subcortical 1/nT2*

*****Left Accumbens*****

| **Variable** | **Beta** | **95% CI** | **p-value** | **q-value**^1^ |
| --- | --- | --- | --- | --- |
| Proportion of concentrate days ratio (log) | -0.01 | -0.03, 0.02 | 0.5 | >0.9 |
| Proportion of flower days ratio (log) | -0.01 | -0.03, 0.02 | 0.6 | >0.9 |
| Proportion of nicotine days (log) | 0.00 | -0.03, 0.03 | >0.9 | >0.9 |
| Proportion of standard drinks (log) | -0.02 | -0.05, 0.01 | 0.2 | >0.9 |
| Sex | 0.00 | -0.02, 0.02 | >0.9 | >0.9 |
| Age | 0.01 | -0.01, 0.02 | 0.3 | >0.9 |
| PLE | 0.00 | 0.00, 0.00 | 0.8 | >0.9 |
| ^1^False discovery rate correction for multiple testing | | | | |
| CI = Confidence Interval; PLE = psychotic-like experiences | | | | |

*****Right Accumbens*****

| **Variable** | **Beta** | **95% CI** | **p-value** | **q-value**^1^ |
| --- | --- | --- | --- | --- |
| Proportion of concentrate days ratio (log) | -0.01 | -0.03, 0.01 | 0.4 | 0.6 |
| Proportion of flower days ratio (log) | -0.01 | -0.03, 0.00 | 0.14 | 0.6 |
| Proportion of nicotine days (log) | -0.01 | -0.03, 0.01 | 0.5 | 0.8 |
| Proportion of standard drinks (log) | -0.02 | -0.04, 0.01 | 0.3 | 0.6 |
| Sex | 0.00 | -0.02, 0.02 | >0.9 | >0.9 |
| Age | 0.01 | 0.00, 0.02 | 0.2 | 0.6 |
| PLE | 0.00 | 0.00, 0.00 | 0.8 | 0.9 |
| ^1^False discovery rate correction for multiple testing | | | | |
| CI = Confidence Interval; PLE = psychotic-like experiences | | | | |

*****Left Pallidum*****

| **Variable** | **Beta** | **95% CI** | **p-value** | **q-value**^1^ |
| --- | --- | --- | --- | --- |
| Proportion of concentrate days ratio (log) | -0.05 | -0.09, 0.00 | 0.055 | 0.4 |
| Proportion of flower days ratio (log) | -0.03 | -0.07, 0.02 | 0.2 | 0.4 |
| Proportion of nicotine days (log) | 0.01 | -0.04, 0.06 | 0.8 | 0.8 |
| Proportion of standard drinks (log) | 0.05 | -0.01, 0.12 | 0.13 | 0.4 |
| Sex | -0.01 | -0.06, 0.04 | 0.6 | 0.7 |
| Age | 0.02 | -0.01, 0.04 | 0.2 | 0.4 |
| PLE | 0.00 | 0.00, 0.00 | 0.5 | 0.7 |
| ^1^False discovery rate correction for multiple testing | | | | |
| CI = Confidence Interval; PLE = psychotic-like experiences | | | | |

*****Right Pallidum*****

| **Variable** | **Beta** | **95% CI** | **p-value** | **q-value**^1^ |
| --- | --- | --- | --- | --- |
| Proportion of concentrate days ratio (log) | -0.04 | -0.08, 0.01 | 0.10 | 0.3 |
| Proportion of flower days ratio (log) | -0.01 | -0.05, 0.03 | 0.7 | 0.8 |
| Proportion of nicotine days (log) | -0.01 | -0.06, 0.04 | 0.7 | 0.8 |
| Proportion of standard drinks (log) | 0.05 | -0.01, 0.11 | 0.11 | 0.3 |
| Sex | 0.00 | -0.05, 0.05 | >0.9 | >0.9 |
| Age | 0.02 | 0.00, 0.04 | 0.057 | 0.3 |
| PLE | 0.00 | 0.00, 0.00 | 0.7 | 0.8 |
| ^1^False discovery rate correction for multiple testing | | | | |
| CI = Confidence Interval; PLE = psychotic-like experiences | | | | |

*****Left Caudate*****

| **Variable** | **Beta** | **95% CI** | **p-value** | **q-value**^1^ |
| --- | --- | --- | --- | --- |
| Proportion of concentrate days ratio (log) | -0.02 | -0.03, 0.00 | 0.062 | 0.4 |
| Proportion of flower days ratio (log) | 0.01 | -0.01, 0.02 | 0.4 | 0.7 |
| Proportion of nicotine days (log) | -0.01 | -0.03, 0.01 | 0.3 | 0.7 |
| Proportion of standard drinks (log) | -0.01 | -0.03, 0.02 | 0.6 | 0.8 |
| Sex | 0.00 | -0.02, 0.01 | 0.8 | 0.8 |
| Age | 0.00 | 0.00, 0.01 | 0.4 | 0.7 |
| PLE | 0.00 | 0.00, 0.00 | 0.7 | 0.8 |
| ^1^False discovery rate correction for multiple testing | | | | |
| CI = Confidence Interval; PLE = psychotic-like experiences | | | | |

*****Right Caudate*****

| **Variable** | **Beta** | **95% CI** | **p-value** | **q-value**^1^ |
| --- | --- | --- | --- | --- |
| Proportion of concentrate days ratio (log) | -0.01 | -0.03, 0.01 | 0.2 | 0.9 |
| Proportion of flower days ratio (log) | 0.00 | -0.01, 0.02 | 0.6 | 0.9 |
| Proportion of nicotine days (log) | -0.01 | -0.03, 0.01 | 0.3 | 0.9 |
| Proportion of standard drinks (log) | -0.01 | -0.03, 0.02 | 0.6 | 0.9 |
| Sex | 0.00 | -0.02, 0.02 | >0.9 | >0.9 |
| Age | 0.00 | -0.01, 0.01 | 0.7 | 0.9 |
| PLE | 0.00 | 0.00, 0.00 | 0.7 | 0.9 |
| ^1^False discovery rate correction for multiple testing | | | | |
| CI = Confidence Interval; PLE = psychotic-like experiences | | | | |

*****Left Putamen*****

| **Variable** | **Beta** | **95% CI** | **p-value** | **q-value**^1^ |
| --- | --- | --- | --- | --- |
| Proportion of concentrate days ratio (log) | -0.01 | -0.03, 0.00 | 0.11 | 0.7 |
| Proportion of flower days ratio (log) | -0.01 | -0.02, 0.01 | 0.3 | 0.7 |
| Proportion of nicotine days (log) | -0.01 | -0.02, 0.01 | 0.4 | 0.7 |
| Proportion of standard drinks (log) | 0.00 | -0.02, 0.02 | 0.9 | 0.9 |
| Sex | -0.01 | -0.02, 0.01 | 0.4 | 0.7 |
| Age | 0.00 | -0.01, 0.01 | 0.6 | 0.7 |
| PLE | 0.00 | 0.00, 0.00 | 0.5 | 0.7 |
| ^1^False discovery rate correction for multiple testing | | | | |
| CI = Confidence Interval; PLE = psychotic-like experiences | | | | |

*****Right Putamen*****

| **Variable** | **Beta** | **95% CI** | **p-value** | **q-value**^1^ |
| --- | --- | --- | --- | --- |
| Proportion of concentrate days ratio (log) | 0.00 | -0.02, 0.01 | 0.8 | 0.8 |
| Proportion of flower days ratio (log) | -0.01 | -0.02, 0.01 | 0.3 | 0.7 |
| Proportion of nicotine days (log) | -0.01 | -0.02, 0.01 | 0.5 | 0.7 |
| Proportion of standard drinks (log) | 0.01 | -0.02, 0.03 | 0.6 | 0.7 |
| Sex | 0.01 | -0.01, 0.02 | 0.3 | 0.7 |
| Age | 0.01 | 0.00, 0.01 | 0.2 | 0.7 |
| PLE | 0.00 | 0.00, 0.00 | 0.5 | 0.7 |
| ^1^False discovery rate correction for multiple testing | | | | |
| CI = Confidence Interval; PLE = psychotic-like experiences | | | | |

*****Left Thalamus*****

| **Variable** | **Beta** | **95% CI** | **p-value** | **q-value**^1^ |
| --- | --- | --- | --- | --- |
| Proportion of concentrate days ratio (log) | -0.01 | -0.02, 0.00 | 0.15 | 0.3 |
| Proportion of flower days ratio (log) | 0.00 | -0.02, 0.01 | 0.4 | 0.4 |
| Proportion of nicotine days (log) | 0.01 | -0.01, 0.02 | 0.3 | 0.4 |
| Proportion of standard drinks (log) | -0.02 | -0.03, 0.00 | 0.057 | 0.3 |
| Sex | -0.01 | -0.02, 0.01 | 0.4 | 0.4 |
| Age | 0.00 | 0.00, 0.01 | 0.2 | 0.3 |
| PLE | 0.00 | 0.00, 0.00 | 0.13 | 0.3 |
| ^1^False discovery rate correction for multiple testing | | | | |
| CI = Confidence Interval; PLE = psychotic-like experiences | | | | |

*****Right Thalamus*****

| **Variable** | **Beta** | **95% CI** | **p-value** | **q-value**^1^ |
| --- | --- | --- | --- | --- |
| Proportion of concentrate days ratio (log) | 0.00 | -0.02, 0.01 | 0.5 | 0.7 |
| Proportion of flower days ratio (log) | 0.00 | -0.02, 0.01 | 0.6 | 0.7 |
| Proportion of nicotine days (log) | 0.00 | -0.02, 0.01 | 0.7 | 0.7 |
| Proportion of standard drinks (log) | -0.01 | -0.03, 0.00 | 0.14 | 0.4 |
| Sex | 0.00 | -0.01, 0.02 | 0.5 | 0.7 |
| Age | 0.00 | 0.00, 0.01 | 0.2 | 0.4 |
| PLE | 0.00 | 0.00, 0.00 | 0.079 | 0.4 |
| ^1^False discovery rate correction for multiple testing | | | | |
| CI = Confidence Interval; PLE = psychotic-like experiences | | | | |

*****Ventral Tegmental Area*****

| **Variable** | **Beta** | **95% CI** | **p-value** | **q-value**^1^ |
| --- | --- | --- | --- | --- |
| Proportion of concentrate days ratio (log) | -0.02 | -0.04, 0.00 | 0.031 | 0.11 |
| Proportion of flower days ratio (log) | 0.01 | -0.01, 0.02 | 0.6 | 0.8 |
| Proportion of nicotine days (log) | 0.00 | -0.02, 0.02 | >0.9 | >0.9 |
| Proportion of standard drinks (log) | -0.01 | -0.04, 0.02 | 0.4 | 0.7 |
| Sex | -0.03 | -0.05, -0.01 | 0.002 | 0.015 |
| Age | 0.01 | 0.00, 0.02 | 0.2 | 0.4 |
| PLE | 0.00 | 0.00, 0.00 | 0.8 | >0.9 |
| ^1^False discovery rate correction for multiple testing | | | | |
| CI = Confidence Interval; PLE = psychotic-like experiences | | | | |

**Table S12.** The association between cannabis frequency 30 days prior to 1/nT2* measurement and subcortical 1/nT2* signal

|  | (n = 78) | | |
| --- | --- | --- | --- |
| *Predictors* | *Estimates* | *CI (95%)* | *p* |
| Intercept | 0.85 | 0.72 – 0.98 | **<0.001** |
| Cannabis sessions past 30-day (log) | -0.01 | -0.01 – < -0.01 | **0.027** |
| Nicotine days past 30-day (log) | < -0.01 | -0.01 – 0.01 | 0.710 |
| Standard drinks past 30-day (log) | < -0.01 | -0.01 – 0.00 | 0.447 |
| Sex | < -0.01 | -0.02 – 0.01 | 0.472 |
| Age | 0.01 | 0.00 – 0.01 | **0.020** |
| PLE | < -0.01 | < -0.01 – 0.00 | 0.725 |

Note. CI = Confidence Interval; PLE = psychotic-like experiences

**Table S13.** Post-hoc models specifying the link between cannabis frequency 30 days prior to 1/nT2* measurement and subcortical 1/nT2* signal intensity

*****Left Accumbens*****

| **Variable** | **Beta** | **95% CI** | **p-value** | **q-value**^1^ |
| --- | --- | --- | --- | --- |
| Cannabis sessions past 30-day (log) | -0.01 | -0.02, 0.00 | 0.3 | 0.8 |
| Nicotine days past 30-day (log) | 0.00 | -0.01, 0.01 | 0.8 | >0.9 |
| Standard drinks past 30-day (log) | -0.01 | -0.02, 0.01 | 0.4 | 0.8 |
| Sex | 0.00 | -0.02, 0.02 | >0.9 | >0.9 |
| Age | 0.00 | -0.01, 0.02 | 0.4 | 0.8 |
| PLE | 0.00 | 0.00, 0.00 | 0.6 | >0.9 |
| ^1^False discovery rate correction for multiple testing | | | | |
| CI = Confidence Interval; PLE = psychotic-like experiences | | | | |

*****Right Accumbens*****

| **Variable** | **Beta** | **95% CI** | **p-value** | **q-value**^1^ |
| --- | --- | --- | --- | --- |
| Cannabis sessions past 30-day (log) | 0.00 | -0.01, 0.00 | 0.3 | 0.6 |
| Nicotine days past 30-day (log) | -0.01 | -0.02, 0.01 | 0.3 | 0.6 |
| Standard drinks past 30-day (log) | -0.01 | -0.02, 0.01 | 0.3 | 0.6 |
| Sex | 0.00 | -0.02, 0.02 | >0.9 | >0.9 |
| Age | 0.00 | -0.01, 0.01 | 0.4 | 0.6 |
| PLE | 0.00 | 0.00, 0.00 | 0.5 | 0.6 |
| ^1^False discovery rate correction for multiple testing | | | | |
| CI = Confidence Interval; PLE = psychotic-like experiences | | | | |

*****Left Pallidum*****

| **Variable** | **Beta** | **95% CI** | **p-value** | **q-value**^1^ |
| --- | --- | --- | --- | --- |
| Cannabis sessions past 30-day (log) | -0.02 | -0.04, 0.00 | 0.10 | 0.3 |
| Nicotine days past 30-day (log) | 0.00 | -0.03, 0.03 | >0.9 | >0.9 |
| Standard drinks past 30-day (log) | 0.00 | -0.02, 0.03 | 0.8 | >0.9 |
| Sex | -0.01 | -0.06, 0.03 | 0.6 | >0.9 |
| Age | 0.02 | 0.00, 0.04 | 0.051 | 0.3 |
| PLE | 0.00 | 0.00, 0.00 | 0.7 | >0.9 |
| ^1^False discovery rate correction for multiple testing | | | | |
| CI = Confidence Interval; PLE = psychotic-like experiences | | | | |

*****Right Pallidum*****

| **Variable** | **Beta** | **95% CI** | **p-value** | **q-value**^1^ |
| --- | --- | --- | --- | --- |
| Cannabis sessions past 30-day (log) | -0.01 | -0.03, 0.01 | 0.3 | >0.9 |
| Nicotine days past 30-day (log) | 0.00 | -0.03, 0.02 | 0.8 | >0.9 |
| Standard drinks past 30-day (log) | 0.00 | -0.03, 0.02 | >0.9 | >0.9 |
| Sex | 0.00 | -0.05, 0.04 | 0.9 | >0.9 |
| Age | 0.03 | 0.01, 0.05 | 0.011 | 0.066 |
| PLE | 0.00 | 0.00, 0.00 | >0.9 | >0.9 |
| ^1^False discovery rate correction for multiple testing | | | | |
| CI = Confidence Interval; PLE = psychotic-like experiences | | | | |

*****Left Caudate*****

| **Variable** | **Beta** | **95% CI** | **p-value** | **q-value**^1^ |
| --- | --- | --- | --- | --- |
| Cannabis sessions past 30-day (log) | 0.00 | -0.01, 0.01 | 0.6 | >0.9 |
| Nicotine days past 30-day (log) | 0.00 | -0.01, 0.00 | 0.2 | >0.9 |
| Standard drinks past 30-day (log) | 0.00 | -0.01, 0.00 | 0.3 | >0.9 |
| Sex | 0.00 | -0.02, 0.02 | >0.9 | >0.9 |
| Age | 0.00 | -0.01, 0.01 | 0.6 | >0.9 |
| PLE | 0.00 | 0.00, 0.00 | >0.9 | >0.9 |
| ^1^False discovery rate correction for multiple testing | | | | |
| CI = Confidence Interval; PLE = psychotic-like experiences | | | | |

*****Right Caudate*****

| **Variable** | **Beta** | **95% CI** | **p-value** | **q-value**^1^ |
| --- | --- | --- | --- | --- |
| Cannabis sessions past 30-day (log) | 0.00 | -0.01, 0.01 | 0.8 | >0.9 |
| Nicotine days past 30-day (log) | -0.01 | -0.01, 0.00 | 0.2 | 0.7 |
| Standard drinks past 30-day (log) | -0.01 | -0.01, 0.00 | 0.2 | 0.7 |
| Sex | 0.00 | -0.02, 0.02 | >0.9 | >0.9 |
| Age | 0.00 | -0.01, 0.01 | >0.9 | >0.9 |
| PLE | 0.00 | 0.00, 0.00 | >0.9 | >0.9 |
| ^1^False discovery rate correction for multiple testing | | | | |
| CI = Confidence Interval; PLE = psychotic-like experiences | | | | |

*****Left Putamen*****

| **Variable** | **Beta** | **95% CI** | **p-value** | **q-value**^1^ |
| --- | --- | --- | --- | --- |
| Cannabis sessions past 30-day (log) | -0.01 | -0.01, 0.00 | 0.013 | 0.077 |
| Nicotine days past 30-day (log) | 0.00 | -0.01, 0.01 | 0.7 | 0.8 |
| Standard drinks past 30-day (log) | 0.00 | -0.01, 0.01 | >0.9 | >0.9 |
| Sex | -0.01 | -0.02, 0.01 | 0.3 | 0.8 |
| Age | 0.00 | -0.01, 0.01 | 0.6 | 0.8 |
| PLE | 0.00 | 0.00, 0.00 | 0.6 | 0.8 |
| ^1^False discovery rate correction for multiple testing | | | | |
| CI = Confidence Interval; PLE = psychotic-like experiences | | | | |

*****Right Putamen*****

| **Variable** | **Beta** | **95% CI** | **p-value** | **q-value**^1^ |
| --- | --- | --- | --- | --- |
| Cannabis sessions past 30-day (log) | 0.00 | -0.01, 0.00 | 0.2 | 0.7 |
| Nicotine days past 30-day (log) | 0.00 | -0.01, 0.01 | 0.6 | 0.7 |
| Standard drinks past 30-day (log) | 0.00 | -0.01, 0.01 | 0.7 | 0.7 |
| Sex | 0.01 | -0.01, 0.02 | 0.4 | 0.7 |
| Age | 0.01 | 0.00, 0.01 | 0.11 | 0.6 |
| PLE | 0.00 | 0.00, 0.00 | 0.5 | 0.7 |
| ^1^False discovery rate correction for multiple testing | | | | |
| CI = Confidence Interval; PLE = psychotic-like experiences | | | | |

*****Left Thalamus*****

| **Variable** | **Beta** | **95% CI** | **p-value** | **q-value**^1^ |
| --- | --- | --- | --- | --- |
| Cannabis sessions past 30-day (log) | 0.00 | -0.01, 0.00 | 0.064 | 0.2 |
| Nicotine days past 30-day (log) | 0.00 | 0.00, 0.01 | 0.5 | 0.5 |
| Standard drinks past 30-day (log) | 0.00 | -0.01, 0.00 | 0.2 | 0.3 |
| Sex | 0.00 | -0.02, 0.01 | 0.5 | 0.5 |
| Age | 0.00 | 0.00, 0.01 | 0.4 | 0.5 |
| PLE | 0.00 | 0.00, 0.00 | 0.056 | 0.2 |
| ^1^False discovery rate correction for multiple testing | | | | |
| CI = Confidence Interval; PLE = psychotic-like experiences | | | | |

*****Right Thalamus*****

| **Variable** | **Beta** | **95% CI** | **p-value** | **q-value**^1^ |
| --- | --- | --- | --- | --- |
| Cannabis sessions past 30-day (log) | 0.00 | -0.01, 0.00 | 0.088 | 0.3 |
| Nicotine days past 30-day (log) | 0.00 | -0.01, 0.01 | >0.9 | >0.9 |
| Standard drinks past 30-day (log) | 0.00 | -0.01, 0.00 | 0.2 | 0.4 |
| Sex | 0.00 | -0.01, 0.02 | 0.6 | 0.7 |
| Age | 0.00 | 0.00, 0.01 | 0.3 | 0.4 |
| PLE | 0.00 | 0.00, 0.00 | 0.046 | 0.3 |
| ^1^False discovery rate correction for multiple testing | | | | |
| CI = Confidence Interval; PLE = psychotic-like experiences | | | | |

*****Ventral Tegmental Area*****

| **Variable** | **Beta** | **95% CI** | **p-value** | **q-value**^1^ |
| --- | --- | --- | --- | --- |
| Cannabis sessions past 30-day (log) | -0.01 | -0.02, 0.00 | 0.055 | 0.2 |
| Nicotine days past 30-day (log) | 0.00 | -0.01, 0.01 | 0.4 | 0.5 |
| Standard drinks past 30-day (log) | 0.00 | -0.01, 0.01 | 0.5 | 0.5 |
| Sex | -0.03 | -0.05, -0.01 | 0.002 | 0.011 |
| Age | 0.01 | 0.00, 0.01 | 0.2 | 0.5 |
| PLE | 0.00 | 0.00, 0.00 | 0.5 | 0.5 |
| ^1^False discovery rate correction for multiple testing | | | | |
| CI = Confidence Interval; PLE = psychotic-like experiences | | | | |

**Table S14.** The association between lifetime concentrated cannabis and smoked grams of cannabis and subcortical 1/nT2* signal intensity

|  | (n = 76) | | |
| --- | --- | --- | --- |
| *Predictors* | *Estimates* | *CI (95%)* | *p* |
| Intercept | 0.84 | 0.71 – 0.98 | **<0.001** |
| Lifetime cannabis grams (log) | < -0.01 | -0.01 – 0.01 | 0.660 |
| Lifetime concentrate times (log) | < -0.01 | -0.01 – 0.00 | 0.115 |
| Lifetime e-cig times (log) | 0.00 | < -0.01 – 0.00 | 0.913 |
| Lifetime cigarettes (log) | -0.01 | -0.01 – 0.00 | 0.237 |
| Lifetime standard drinks (log) | 0.00 | -0.01 – 0.01 | 0.939 |
| Sex | < -0.01 | -0.02 – 0.01 | 0.458 |
| Age | 0.01 | < -0.01 – 0.01 | 0.061 |
| PLE | 0.00 | < -0.01 – 0.00 | 0.795 |

Note. CI = Confidence Interval; PLE = psychotic-like experiences

**Table S15.** The association between cannabis quantity (models 1 and 2) and subjective intoxication (model 3) with subcortical 1/nT2* signal intensity among adolescents with no nicotine use during the Timeline Followback measurement period

|  | **Model 1** (n = 53) | | | **Model 2** (n = 53) | | | **Model 3** (n = 53) | | |
| --- | --- | --- | --- | --- | --- | --- | --- | --- | --- |
| *Predictors* | *Estimates* | *CI (95%)* | *p* | *Estimates* | *CI (95%)* | *p* | *Estimates* | *CI (95%)* | *p* |
| Intercept | 0.90 | 0.72 – 1.08 | **<0.001** | 0.88 | 0.69 – 1.07 | **<0.001** | 0.91 | 0.73 – 1.08 | **<0.001** |
| Daily concentrate hits (log) | -0.01 | -0.02 – < -0.01 | **0.003** |  |  |  |  |  |  |
| Proportion of standard drinks (log) | 0.02 | -0.01 – 0.04 | 0.183 | 0.01 | -0.01 – 0.04 | 0.278 | 0.02 | -0.00 – 0.05 | 0.079 |
| Sex | -0.01 | -0.02 – 0.01 | 0.445 | -0.01 | -0.02 – 0.01 | 0.470 | -0.01 | -0.03 – 0.01 | 0.240 |
| Age | 0.01 | -0.00 – 0.01 | 0.162 | 0.01 | -0.00 – 0.01 | 0.188 | 0.01 | -0.00 – 0.01 | 0.143 |
| PLE | -0.00 | -0.00 – 0.00 | 0.965 | -0.00 | -0.00 – 0.00 | 0.831 | -0.00 | -0.00 – 0.00 | 0.621 |
| Daily cannabis grams (log) |  |  |  | -0.02 | -0.04 – < -0.01 | **0.027** |  |  |  |
| Daily hours high ratio (log) |  |  |  |  |  |  | -0.02 | -0.03 – -0.01 | **0.001** |

Note. CI = Confidence Interval; PLE = psychotic-like experiences

**Table S16.** The association between cannabis frequency (model 1) and cannabis use disorder severity (model 2) with subcortical 1/nT2* signal intensity among adolescents with no nicotine use during the Timeline Followback measurement period

|  | **Model 1** (n = 53) | | | **Model 2** (n = 53) | | |
| --- | --- | --- | --- | --- | --- | --- |
| *Predictors* | *Estimates* | *CI (95%)* | *p* | *Estimates* | *CI (95%)* | *p* |
| Intercept | 0.90 | 0.73 – 1.08 | **<0.001** | 0.91 | 0.73 – 1.10 | **<0.001** |
| Cannabis sessions ratio (log) | -0.03 | -0.04 – -0.01 | **0.001** |  |  |  |
| Proportion of standard drinks (log) | 0.02 | < -0.01 – 0.05 | 0.071 | 0.01 | -0.01 – 0.04 | 0.382 |
| Sex | -0.01 | -0.03 – 0.01 | 0.230 | -0.01 | -0.02 – 0.01 | 0.530 |
| Age | 0.01 | < -0.01 – 0.01 | 0.123 | 0.01 | < -0.01 – 0.02 | 0.100 |
| PLE | < -0.01 | < -0.01 – 0.00 | 0.470 | < -0.01 | < -0.01 – 0.00 | 0.593 |
| CUD severity |  |  |  | -0.01 | -0.02 – < -0.01 | **0.012** |

Note. CI = Confidence Interval; CUD = cannabis use disorder; PLE = psychotic-like experiences

**Table S17.** Post-hoc models specifying the link between daily concentrate hits and subcortical 1/nT2* among adolescents with no nicotine use during the Timeline Followback measurement period

*****Left Accumbens*****

| **Variable** | **Beta** | **95% CI** | **p-value** | **q-value**^1^ |
| --- | --- | --- | --- | --- |
| Daily concentrate hits (log) | -0.01 | -0.02, 0.01 | 0.5 | 0.8 |
| Proportion of standard drinks (log) | -0.02 | -0.07, 0.03 | 0.4 | 0.8 |
| Sex | 0.00 | -0.03, 0.03 | >0.9 | >0.9 |
| Age | 0.01 | 0.00, 0.03 | 0.2 | 0.8 |
| PLE | 0.00 | 0.00, 0.00 | 0.6 | 0.8 |
| ^1^False discovery rate correction for multiple testing | | | | |
| CI = Confidence Interval; PLE = psychotic-like experiences | | | | |

*****Right Accumbens*****

| **Variable** | **Beta** | **95% CI** | **p-value** | **q-value**^1^ |
| --- | --- | --- | --- | --- |
| Daily concentrate hits (log) | 0.00 | -0.02, 0.01 | 0.5 | 0.7 |
| Proportion of standard drinks (log) | -0.01 | -0.05, 0.03 | 0.7 | 0.7 |
| Sex | 0.01 | -0.02, 0.04 | 0.5 | 0.7 |
| Age | 0.01 | -0.01, 0.02 | 0.2 | 0.7 |
| PLE | 0.00 | 0.00, 0.00 | 0.7 | 0.7 |
| ^1^False discovery rate correction for multiple testing | | | | |
| CI = Confidence Interval; PLE = psychotic-like experiences | | | | |

*****Left Pallidum*****

| **Variable** | **Beta** | **95% CI** | **p-value** | **q-value**^1^ |
| --- | --- | --- | --- | --- |
| Daily concentrate hits (log) | -0.03 | -0.06, 0.00 | 0.044 | 0.11 |
| Proportion of standard drinks (log) | 0.10 | 0.01, 0.19 | 0.034 | 0.11 |
| Sex | -0.01 | -0.07, 0.05 | 0.7 | 0.7 |
| Age | 0.01 | -0.02, 0.04 | 0.7 | 0.7 |
| PLE | 0.00 | 0.00, 0.00 | 0.6 | 0.7 |
| ^1^False discovery rate correction for multiple testing | | | | |
| CI = Confidence Interval; PLE = psychotic-like experiences | | | | |

*****Right Pallidum*****

| **Variable** | **Beta** | **95% CI** | **p-value** | **q-value**^1^ |
| --- | --- | --- | --- | --- |
| Daily concentrate hits (log) | -0.03 | -0.05, 0.00 | 0.043 | 0.11 |
| Proportion of standard drinks (log) | 0.10 | 0.01, 0.19 | 0.023 | 0.11 |
| Sex | 0.00 | -0.06, 0.06 | >0.9 | >0.9 |
| Age | 0.01 | -0.01, 0.04 | 0.3 | 0.6 |
| PLE | 0.00 | 0.00, 0.00 | 0.8 | >0.9 |
| ^1^False discovery rate correction for multiple testing | | | | |
| CI = Confidence Interval; PLE = psychotic-like experiences | | | | |

*****Left Caudate*****

| **Variable** | **Beta** | **95% CI** | **p-value** | **q-value**^1^ |
| --- | --- | --- | --- | --- |
| Daily concentrate hits (log) | -0.01 | -0.02, 0.00 | 0.030 | 0.15 |
| Proportion of standard drinks (log) | 0.01 | -0.02, 0.04 | 0.5 | 0.6 |
| Sex | -0.01 | -0.03, 0.01 | 0.4 | 0.6 |
| Age | 0.01 | -0.01, 0.02 | 0.3 | 0.6 |
| PLE | 0.00 | 0.00, 0.00 | 0.8 | 0.8 |
| ^1^False discovery rate correction for multiple testing | | | | |
| CI = Confidence Interval; PLE = psychotic-like experiences | | | | |

*****Right Caudate*****

| **Variable** | **Beta** | **95% CI** | **p-value** | **q-value**^1^ |
| --- | --- | --- | --- | --- |
| Daily concentrate hits (log) | -0.01 | -0.02, 0.00 | 0.013 | 0.066 |
| Proportion of standard drinks (log) | 0.01 | -0.02, 0.05 | 0.3 | 0.6 |
| Sex | -0.01 | -0.03, 0.01 | 0.5 | 0.6 |
| Age | 0.01 | 0.00, 0.02 | 0.2 | 0.6 |
| PLE | 0.00 | 0.00, 0.00 | 0.9 | 0.9 |
| ^1^False discovery rate correction for multiple testing | | | | |
| CI = Confidence Interval; PLE = psychotic-like experiences | | | | |

*****Left Putamen*****

| **Variable** | **Beta** | **95% CI** | **p-value** | **q-value**^1^ |
| --- | --- | --- | --- | --- |
| Daily concentrate hits (log) | -0.01 | -0.02, 0.00 | 0.11 | 0.5 |
| Proportion of standard drinks (log) | 0.00 | -0.03, 0.03 | 0.9 | 0.9 |
| Sex | -0.01 | -0.03, 0.01 | 0.3 | 0.8 |
| Age | 0.00 | -0.01, 0.01 | 0.7 | 0.9 |
| PLE | 0.00 | 0.00, 0.00 | 0.7 | 0.9 |
| ^1^False discovery rate correction for multiple testing | | | | |
| CI = Confidence Interval; PLE = psychotic-like experiences | | | | |

*****Right Putamen*****

| **Variable** | **Beta** | **95% CI** | **p-value** | **q-value**^1^ |
| --- | --- | --- | --- | --- |
| Daily concentrate hits (log) | 0.00 | -0.01, 0.01 | 0.6 | 0.7 |
| Proportion of standard drinks (log) | 0.01 | -0.02, 0.04 | 0.6 | 0.7 |
| Sex | 0.01 | -0.01, 0.03 | 0.6 | 0.7 |
| Age | 0.00 | -0.01, 0.01 | 0.8 | 0.8 |
| PLE | 0.00 | 0.00, 0.00 | 0.5 | 0.7 |
| ^1^False discovery rate correction for multiple testing | | | | |
| CI = Confidence Interval; PLE = psychotic-like experiences | | | | |

*****Left Thalamus*****

| **Variable** | **Beta** | **95% CI** | **p-value** | **q-value**^1^ |
| --- | --- | --- | --- | --- |
| Daily concentrate hits (log) | -0.01 | -0.01, 0.00 | 0.068 | 0.3 |
| Proportion of standard drinks (log) | -0.01 | -0.04, 0.01 | 0.2 | 0.3 |
| Sex | -0.01 | -0.02, 0.01 | 0.3 | 0.3 |
| Age | 0.00 | -0.01, 0.01 | 0.8 | 0.8 |
| PLE | 0.00 | 0.00, 0.00 | 0.2 | 0.3 |
| ^1^False discovery rate correction for multiple testing | | | | |
| CI = Confidence Interval; PLE = psychotic-like experiences | | | | |

*****Right Thalamus*****

| **Variable** | **Beta** | **95% CI** | **p-value** | **q-value**^1^ |
| --- | --- | --- | --- | --- |
| Daily concentrate hits (log) | 0.00 | -0.01, 0.00 | 0.2 | 0.4 |
| Proportion of standard drinks (log) | -0.01 | -0.04, 0.01 | 0.2 | 0.4 |
| Sex | 0.01 | -0.01, 0.02 | 0.5 | 0.6 |
| Age | 0.00 | -0.01, 0.01 | 0.6 | 0.6 |
| PLE | 0.00 | 0.00, 0.00 | 0.077 | 0.4 |
| ^1^False discovery rate correction for multiple testing | | | | |
| CI = Confidence Interval; PLE = psychotic-like experiences | | | | |

*****Ventral Tegmental Area*****

| **Variable** | **Beta** | **95% CI** | **p-value** | **q-value**^1^ |
| --- | --- | --- | --- | --- |
| Daily concentrate hits (log) | -0.02 | -0.03, -0.01 | <0.001 | <0.001 |
| Proportion of standard drinks (log) | 0.00 | -0.03, 0.04 | 0.9 | >0.9 |
| Sex | -0.05 | -0.07, -0.03 | <0.001 | <0.001 |
| Age | 0.01 | -0.01, 0.02 | 0.3 | 0.6 |
| PLE | 0.00 | 0.00, 0.00 | >0.9 | >0.9 |
| ^1^False discovery rate correction for multiple testing | | | | |
| CI = Confidence Interval; PLE = psychotic-like experiences | | | | |

**Table S18.** Post-hoc models specifying the link between daily cannabis grams and subcortical 1/nT2* among adolescents with no nicotine use during the Timeline Followback measurement period

*****Left Accumbens*****

| **Variable** | **Beta** | **95% CI** | **p-value** | **q-value**^1^ |
| --- | --- | --- | --- | --- |
| Daily cannabis grams (log) | -0.02 | -0.05, 0.01 | 0.3 | 0.7 |
| Proportion of standard drinks (log) | -0.02 | -0.06, 0.03 | 0.5 | 0.9 |
| Sex | 0.00 | -0.04, 0.03 | 0.9 | 0.9 |
| Age | 0.01 | 0.00, 0.03 | 0.15 | 0.7 |
| PLE | 0.00 | 0.00, 0.00 | 0.7 | 0.9 |
| ^1^False discovery rate correction for multiple testing | | | | |
| CI = Confidence Interval; PLE = psychotic-like experiences | | | | |

*****Right Accumbens*****

| **Variable** | **Beta** | **95% CI** | **p-value** | **q-value**^1^ |
| --- | --- | --- | --- | --- |
| Daily cannabis grams (log) | -0.03 | -0.06, 0.00 | 0.038 | 0.2 |
| Proportion of standard drinks (log) | 0.00 | -0.04, 0.04 | >0.9 | >0.9 |
| Sex | 0.00 | -0.03, 0.03 | >0.9 | >0.9 |
| Age | 0.01 | 0.00, 0.02 | 0.2 | 0.4 |
| PLE | 0.00 | 0.00, 0.00 | >0.9 | >0.9 |
| ^1^False discovery rate correction for multiple testing | | | | |
| CI = Confidence Interval; PLE = psychotic-like experiences | | | | |

*****Left Pallidum*****

| **Variable** | **Beta** | **95% CI** | **p-value** | **q-value**^1^ |
| --- | --- | --- | --- | --- |
| Daily cannabis grams (log) | -0.07 | -0.13, -0.01 | 0.033 | 0.083 |
| Proportion of standard drinks (log) | 0.10 | 0.01, 0.19 | 0.030 | 0.083 |
| Sex | -0.02 | -0.08, 0.05 | 0.6 | 0.7 |
| Age | 0.01 | -0.02, 0.04 | 0.7 | 0.7 |
| PLE | 0.00 | 0.00, 0.01 | 0.6 | 0.7 |
| ^1^False discovery rate correction for multiple testing | | | | |
| CI = Confidence Interval; PLE = psychotic-like experiences | | | | |

*****Right Pallidum*****

| **Variable** | **Beta** | **95% CI** | **p-value** | **q-value**^1^ |
| --- | --- | --- | --- | --- |
| Daily cannabis grams (log) | -0.05 | -0.11, 0.02 | 0.14 | 0.4 |
| Proportion of standard drinks (log) | 0.09 | 0.01, 0.18 | 0.036 | 0.2 |
| Sex | 0.00 | -0.06, 0.07 | >0.9 | >0.9 |
| Age | 0.01 | -0.02, 0.04 | 0.3 | 0.6 |
| PLE | 0.00 | 0.00, 0.00 | >0.9 | >0.9 |
| ^1^False discovery rate correction for multiple testing | | | | |
| CI = Confidence Interval; PLE = psychotic-like experiences | | | | |

*****Left Caudate*****

| **Variable** | **Beta** | **95% CI** | **p-value** | **q-value**^1^ |
| --- | --- | --- | --- | --- |
| Daily cannabis grams (log) | 0.00 | -0.03, 0.02 | 0.8 | >0.9 |
| Proportion of standard drinks (log) | 0.00 | -0.03, 0.03 | >0.9 | >0.9 |
| Sex | 0.00 | -0.03, 0.02 | 0.7 | >0.9 |
| Age | 0.01 | -0.01, 0.02 | 0.4 | >0.9 |
| PLE | 0.00 | 0.00, 0.00 | 0.7 | >0.9 |
| ^1^False discovery rate correction for multiple testing | | | | |
| CI = Confidence Interval; PLE = psychotic-like experiences | | | | |

*****Right Caudate*****

| **Variable** | **Beta** | **95% CI** | **p-value** | **q-value**^1^ |
| --- | --- | --- | --- | --- |
| Daily cannabis grams (log) | -0.02 | -0.04, 0.01 | 0.2 | 0.7 |
| Proportion of standard drinks (log) | 0.01 | -0.02, 0.04 | 0.6 | 0.8 |
| Sex | -0.01 | -0.03, 0.02 | 0.7 | 0.8 |
| Age | 0.01 | 0.00, 0.02 | 0.3 | 0.7 |
| PLE | 0.00 | 0.00, 0.00 | 0.9 | 0.9 |
| ^1^False discovery rate correction for multiple testing | | | | |
| CI = Confidence Interval; PLE = psychotic-like experiences | | | | |

*****Left Putamen*****

| **Variable** | **Beta** | **95% CI** | **p-value** | **q-value**^1^ |
| --- | --- | --- | --- | --- |
| Daily cannabis grams (log) | -0.02 | -0.04, 0.00 | 0.13 | 0.6 |
| Proportion of standard drinks (log) | 0.00 | -0.03, 0.03 | 0.9 | 0.9 |
| Sex | -0.01 | -0.03, 0.01 | 0.3 | 0.7 |
| Age | 0.00 | -0.01, 0.01 | 0.7 | 0.9 |
| PLE | 0.00 | 0.00, 0.00 | 0.6 | 0.9 |
| ^1^False discovery rate correction for multiple testing | | | | |
| CI = Confidence Interval; PLE = psychotic-like experiences | | | | |

*****Right Putamen*****

| **Variable** | **Beta** | **95% CI** | **p-value** | **q-value**^1^ |
| --- | --- | --- | --- | --- |
| Daily cannabis grams (log) | 0.00 | -0.02, 0.02 | >0.9 | >0.9 |
| Proportion of standard drinks (log) | 0.01 | -0.02, 0.04 | 0.7 | >0.9 |
| Sex | 0.01 | -0.01, 0.03 | 0.5 | >0.9 |
| Age | 0.00 | -0.01, 0.01 | 0.8 | >0.9 |
| PLE | 0.00 | 0.00, 0.00 | 0.6 | >0.9 |
| ^1^False discovery rate correction for multiple testing | | | | |
| CI = Confidence Interval; PLE = psychotic-like experiences | | | | |

*****Left Thalamus*****

| **Variable** | **Beta** | **95% CI** | **p-value** | **q-value**^1^ |
| --- | --- | --- | --- | --- |
| Daily cannabis grams (log) | -0.01 | -0.02, 0.01 | 0.4 | 0.5 |
| Proportion of standard drinks (log) | -0.02 | -0.04, 0.01 | 0.14 | 0.3 |
| Sex | -0.01 | -0.02, 0.01 | 0.4 | 0.5 |
| Age | 0.00 | -0.01, 0.01 | 0.9 | 0.9 |
| PLE | 0.00 | 0.00, 0.00 | 0.11 | 0.3 |
| ^1^False discovery rate correction for multiple testing | | | | |
| CI = Confidence Interval; PLE = psychotic-like experiences | | | | |

*****Right Thalamus*****

| **Variable** | **Beta** | **95% CI** | **p-value** | **q-value**^1^ |
| --- | --- | --- | --- | --- |
| Daily cannabis grams (log) | 0.00 | -0.02, 0.02 | 0.8 | 0.8 |
| Proportion of standard drinks (log) | -0.02 | -0.05, 0.01 | 0.13 | 0.3 |
| Sex | 0.01 | -0.01, 0.03 | 0.3 | 0.5 |
| Age | 0.00 | -0.01, 0.01 | 0.7 | 0.8 |
| PLE | 0.00 | 0.00, 0.00 | 0.032 | 0.2 |
| ^1^False discovery rate correction for multiple testing | | | | |
| CI = Confidence Interval; PLE = psychotic-like experiences | | | | |

*****Ventral Tegmental Area*****

| **Variable** | **Beta** | **95% CI** | **p-value** | **q-value**^1^ |
| --- | --- | --- | --- | --- |
| Daily cannabis grams (log) | -0.03 | -0.05, 0.00 | 0.052 | 0.13 |
| Proportion of standard drinks (log) | -0.01 | -0.04, 0.03 | 0.7 | 0.7 |
| Sex | -0.05 | -0.07, -0.02 | <0.001 | 0.005 |
| Age | 0.00 | -0.01, 0.02 | 0.4 | 0.7 |
| PLE | 0.00 | 0.00, 0.00 | 0.6 | 0.7 |
| ^1^False discovery rate correction for multiple testing | | | | |
| CI = Confidence Interval; PLE = psychotic-like experiences | | | | |

**Table S19.** Post-hoc models specifying the link between hours high and subcortical 1/nT2* among adolescents with no nicotine use during the Timeline Followback measurement period

*****Left Accumbens*****

| **Variable** | **Beta** | **95% CI** | **p-value** | **q-value**^1^ |
| --- | --- | --- | --- | --- |
| Daily hours high ratio (log) | -0.02 | -0.04, 0.01 | 0.2 | 0.5 |
| Proportion of standard drinks (log) | -0.01 | -0.06, 0.04 | 0.7 | 0.8 |
| Sex | 0.00 | -0.04, 0.03 | 0.8 | 0.8 |
| Age | 0.01 | 0.00, 0.03 | 0.14 | 0.5 |
| PLE | 0.00 | 0.00, 0.00 | 0.6 | 0.8 |
| ^1^False discovery rate correction for multiple testing | | | | |
| CI = Confidence Interval; PLE = psychotic-like experiences | | | | |

*****Right Accumbens*****

| **Variable** | **Beta** | **95% CI** | **p-value** | **q-value**^1^ |
| --- | --- | --- | --- | --- |
| Daily hours high ratio (log) | -0.01 | -0.03, 0.01 | 0.3 | 0.7 |
| Proportion of standard drinks (log) | 0.00 | -0.04, 0.04 | >0.9 | >0.9 |
| Sex | 0.01 | -0.02, 0.03 | 0.7 | 0.9 |
| Age | 0.01 | 0.00, 0.02 | 0.2 | 0.7 |
| PLE | 0.00 | 0.00, 0.00 | 0.7 | 0.9 |
| ^1^False discovery rate correction for multiple testing | | | | |
| CI = Confidence Interval; PLE = psychotic-like experiences | | | | |

*****Left Pallidum*****

| **Variable** | **Beta** | **95% CI** | **p-value** | **q-value**^1^ |
| --- | --- | --- | --- | --- |
| Daily hours high ratio (log) | -0.05 | -0.10, -0.01 | 0.029 | 0.073 |
| Proportion of standard drinks (log) | 0.11 | 0.02, 0.21 | 0.019 | 0.073 |
| Sex | -0.02 | -0.09, 0.04 | 0.5 | 0.8 |
| Age | 0.01 | -0.02, 0.04 | 0.7 | 0.8 |
| PLE | 0.00 | 0.00, 0.00 | 0.8 | 0.8 |
| ^1^False discovery rate correction for multiple testing | | | | |
| CI = Confidence Interval; PLE = psychotic-like experiences | | | | |

*****Right Pallidum*****

| **Variable** | **Beta** | **95% CI** | **p-value** | **q-value**^1^ |
| --- | --- | --- | --- | --- |
| Daily hours high ratio (log) | -0.04 | -0.08, 0.01 | 0.10 | 0.3 |
| Proportion of standard drinks (log) | 0.11 | 0.01, 0.20 | 0.024 | 0.12 |
| Sex | 0.00 | -0.06, 0.06 | >0.9 | >0.9 |
| Age | 0.01 | -0.02, 0.04 | 0.3 | 0.6 |
| PLE | 0.00 | 0.00, 0.00 | 0.9 | >0.9 |
| ^1^False discovery rate correction for multiple testing | | | | |
| CI = Confidence Interval; PLE = psychotic-like experiences | | | | |

*****Left Caudate*****

| **Variable** | **Beta** | **95% CI** | **p-value** | **q-value**^1^ |
| --- | --- | --- | --- | --- |
| Daily hours high ratio (log) | -0.02 | -0.04, -0.01 | 0.006 | 0.032 |
| Proportion of standard drinks (log) | 0.02 | -0.01, 0.05 | 0.2 | 0.3 |
| Sex | -0.01 | -0.04, 0.01 | 0.2 | 0.3 |
| Age | 0.01 | 0.00, 0.02 | 0.3 | 0.3 |
| PLE | 0.00 | 0.00, 0.00 | >0.9 | >0.9 |
| ^1^False discovery rate correction for multiple testing | | | | |
| CI = Confidence Interval; PLE = psychotic-like experiences | | | | |

*****Right Caudate*****

| **Variable** | **Beta** | **95% CI** | **p-value** | **q-value**^1^ |
| --- | --- | --- | --- | --- |
| Daily hours high ratio (log) | -0.02 | -0.04, -0.01 | 0.009 | 0.046 |
| Proportion of standard drinks (log) | 0.02 | -0.01, 0.05 | 0.2 | 0.3 |
| Sex | -0.01 | -0.03, 0.01 | 0.3 | 0.4 |
| Age | 0.01 | 0.00, 0.02 | 0.2 | 0.3 |
| PLE | 0.00 | 0.00, 0.00 | 0.8 | 0.8 |
| ^1^False discovery rate correction for multiple testing | | | | |
| CI = Confidence Interval; PLE = psychotic-like experiences | | | | |

*****Left Putamen*****

| **Variable** | **Beta** | **95% CI** | **p-value** | **q-value**^1^ |
| --- | --- | --- | --- | --- |
| Daily hours high ratio (log) | -0.02 | -0.03, 0.00 | 0.027 | 0.13 |
| Proportion of standard drinks (log) | 0.01 | -0.02, 0.04 | 0.6 | 0.7 |
| Sex | -0.01 | -0.03, 0.01 | 0.2 | 0.4 |
| Age | 0.00 | -0.01, 0.01 | 0.6 | 0.7 |
| PLE | 0.00 | 0.00, 0.00 | 0.7 | 0.7 |
| ^1^False discovery rate correction for multiple testing | | | | |
| CI = Confidence Interval; PLE = psychotic-like experiences | | | | |

*****Right Putamen*****

| **Variable** | **Beta** | **95% CI** | **p-value** | **q-value**^1^ |
| --- | --- | --- | --- | --- |
| Daily hours high ratio (log) | -0.01 | -0.02, 0.01 | 0.4 | 0.8 |
| Proportion of standard drinks (log) | 0.01 | -0.02, 0.04 | 0.5 | 0.8 |
| Sex | 0.00 | -0.02, 0.03 | 0.7 | 0.8 |
| Age | 0.00 | -0.01, 0.01 | 0.8 | 0.8 |
| PLE | 0.00 | 0.00, 0.00 | 0.5 | 0.8 |
| ^1^False discovery rate correction for multiple testing | | | | |
| CI = Confidence Interval; PLE = psychotic-like experiences | | | | |

*****Left Thalamus*****

| **Variable** | **Beta** | **95% CI** | **p-value** | **q-value**^1^ |
| --- | --- | --- | --- | --- |
| Daily hours high ratio (log) | -0.01 | -0.02, 0.00 | 0.10 | 0.2 |
| Proportion of standard drinks (log) | -0.01 | -0.04, 0.01 | 0.3 | 0.4 |
| Sex | -0.01 | -0.03, 0.01 | 0.2 | 0.4 |
| Age | 0.00 | -0.01, 0.01 | 0.8 | 0.8 |
| PLE | 0.00 | 0.00, 0.00 | 0.088 | 0.2 |
| ^1^False discovery rate correction for multiple testing | | | | |
| CI = Confidence Interval; PLE = psychotic-like experiences | | | | |

*****Right Thalamus*****

| **Variable** | **Beta** | **95% CI** | **p-value** | **q-value**^1^ |
| --- | --- | --- | --- | --- |
| Daily hours high ratio (log) | -0.01 | -0.02, 0.01 | 0.3 | 0.5 |
| Proportion of standard drinks (log) | -0.01 | -0.04, 0.01 | 0.3 | 0.5 |
| Sex | 0.01 | -0.01, 0.02 | 0.5 | 0.6 |
| Age | 0.00 | -0.01, 0.01 | 0.6 | 0.6 |
| PLE | 0.00 | 0.00, 0.00 | 0.043 | 0.2 |
| ^1^False discovery rate correction for multiple testing | | | | |
| CI = Confidence Interval; PLE = psychotic-like experiences | | | | |

*****Ventral Tegmental Area*****

| **Variable** | **Beta** | **95% CI** | **p-value** | **q-value**^1^ |
| --- | --- | --- | --- | --- |
| Daily hours high ratio (log) | -0.03 | -0.05, -0.02 | <0.001 | <0.001 |
| Proportion of standard drinks (log) | 0.01 | -0.03, 0.04 | 0.6 | 0.6 |
| Sex | -0.05 | -0.08, -0.03 | <0.001 | <0.001 |
| Age | 0.01 | -0.01, 0.02 | 0.3 | 0.6 |
| PLE | 0.00 | 0.00, 0.00 | 0.5 | 0.6 |
| ^1^False discovery rate correction for multiple testing | | | | |
| CI = Confidence Interval; PLE = psychotic-like experiences | | | | |

**Table S20.** Post-hoc models specifying the link between cannabis frequency and subcortical 1/nT2* among adolescents with no nicotine use during the Timeline Followback measurement period

*****Left Accumbens*****

| **Variable** | **Beta** | **95% CI** | **p-value** | **q-value**^1^ |
| --- | --- | --- | --- | --- |
| Cannabis sessions ratio (log) | -0.02 | -0.05, 0.01 | 0.2 | 0.5 |
| Proportion of standard drinks (log) | -0.01 | -0.06, 0.04 | 0.7 | 0.8 |
| Sex | 0.00 | -0.04, 0.03 | 0.8 | 0.8 |
| Age | 0.01 | 0.00, 0.03 | 0.14 | 0.5 |
| PLE | 0.00 | 0.00, 0.00 | 0.5 | 0.8 |
| ^1^False discovery rate correction for multiple testing | | | | |
| CI = Confidence Interval; PLE = psychotic-like experiences | | | | |

*****Right Accumbens*****

| **Variable** | **Beta** | **95% CI** | **p-value** | **q-value**^1^ |
| --- | --- | --- | --- | --- |
| Cannabis sessions ratio (log) | -0.01 | -0.03, 0.01 | 0.4 | 0.8 |
| Proportion of standard drinks (log) | 0.00 | -0.05, 0.04 | 0.8 | 0.8 |
| Sex | 0.01 | -0.02, 0.04 | 0.6 | 0.8 |
| Age | 0.01 | 0.00, 0.02 | 0.2 | 0.8 |
| PLE | 0.00 | 0.00, 0.00 | 0.6 | 0.8 |
| ^1^False discovery rate correction for multiple testing | | | | |
| CI = Confidence Interval; PLE = psychotic-like experiences | | | | |

*****Left Pallidum*****

| **Variable** | **Beta** | **95% CI** | **p-value** | **q-value**^1^ |
| --- | --- | --- | --- | --- |
| Cannabis sessions ratio (log) | -0.06 | -0.12, -0.01 | 0.023 | 0.057 |
| Proportion of standard drinks (log) | 0.12 | 0.02, 0.21 | 0.017 | 0.057 |
| Sex | -0.02 | -0.09, 0.04 | 0.5 | 0.8 |
| Age | 0.01 | -0.02, 0.04 | 0.6 | 0.8 |
| PLE | 0.00 | 0.00, 0.00 | >0.9 | >0.9 |
| ^1^False discovery rate correction for multiple testing | | | | |
| CI = Confidence Interval; PLE = psychotic-like experiences | | | | |

*****Right Pallidum*****

| **Variable** | **Beta** | **95% CI** | **p-value** | **q-value**^1^ |
| --- | --- | --- | --- | --- |
| Cannabis sessions ratio (log) | -0.05 | -0.10, 0.00 | 0.075 | 0.2 |
| Proportion of standard drinks (log) | 0.11 | 0.02, 0.20 | 0.021 | 0.10 |
| Sex | 0.00 | -0.06, 0.06 | >0.9 | >0.9 |
| Age | 0.01 | -0.01, 0.04 | 0.3 | 0.5 |
| PLE | 0.00 | 0.00, 0.00 | 0.8 | >0.9 |
| ^1^False discovery rate correction for multiple testing | | | | |
| CI = Confidence Interval; PLE = psychotic-like experiences | | | | |

*****Left Caudate*****

| **Variable** | **Beta** | **95% CI** | **p-value** | **q-value**^1^ |
| --- | --- | --- | --- | --- |
| Cannabis sessions ratio (log) | -0.03 | -0.05, -0.01 | 0.002 | 0.011 |
| Proportion of standard drinks (log) | 0.02 | -0.01, 0.05 | 0.2 | 0.3 |
| Sex | -0.02 | -0.04, 0.01 | 0.14 | 0.3 |
| Age | 0.01 | 0.00, 0.02 | 0.2 | 0.3 |
| PLE | 0.00 | 0.00, 0.00 | 0.9 | 0.9 |
| ^1^False discovery rate correction for multiple testing | | | | |
| CI = Confidence Interval; PLE = psychotic-like experiences | | | | |

*****Right Caudate*****

| **Variable** | **Beta** | **95% CI** | **p-value** | **q-value**^1^ |
| --- | --- | --- | --- | --- |
| Cannabis sessions ratio (log) | -0.03 | -0.04, -0.01 | 0.007 | 0.036 |
| Proportion of standard drinks (log) | 0.02 | -0.01, 0.05 | 0.2 | 0.3 |
| Sex | -0.01 | -0.03, 0.01 | 0.3 | 0.4 |
| Age | 0.01 | 0.00, 0.02 | 0.2 | 0.3 |
| PLE | 0.00 | 0.00, 0.00 | 0.7 | 0.7 |
| ^1^False discovery rate correction for multiple testing | | | | |
| CI = Confidence Interval; PLE = psychotic-like experiences | | | | |

*****Left Putamen*****

| **Variable** | **Beta** | **95% CI** | **p-value** | **q-value**^1^ |
| --- | --- | --- | --- | --- |
| Cannabis sessions ratio (log) | -0.02 | -0.04, 0.00 | 0.027 | 0.13 |
| Proportion of standard drinks (log) | 0.01 | -0.02, 0.04 | 0.6 | 0.8 |
| Sex | -0.01 | -0.03, 0.01 | 0.2 | 0.4 |
| Age | 0.00 | -0.01, 0.01 | 0.6 | 0.8 |
| PLE | 0.00 | 0.00, 0.00 | 0.9 | 0.9 |
| ^1^False discovery rate correction for multiple testing | | | | |
| CI = Confidence Interval; PLE = psychotic-like experiences | | | | |

*****Right Putamen*****

| **Variable** | **Beta** | **95% CI** | **p-value** | **q-value**^1^ |
| --- | --- | --- | --- | --- |
| Cannabis sessions ratio (log) | -0.01 | -0.03, 0.01 | 0.4 | 0.8 |
| Proportion of standard drinks (log) | 0.01 | -0.02, 0.04 | 0.5 | 0.8 |
| Sex | 0.00 | -0.02, 0.03 | 0.7 | 0.8 |
| Age | 0.00 | -0.01, 0.01 | 0.8 | 0.8 |
| PLE | 0.00 | 0.00, 0.00 | 0.5 | 0.8 |
| ^1^False discovery rate correction for multiple testing | | | | |
| CI = Confidence Interval; PLE = psychotic-like experiences | | | | |

*****Left Thalamus*****

| **Variable** | **Beta** | **95% CI** | **p-value** | **q-value**^1^ |
| --- | --- | --- | --- | --- |
| Cannabis sessions ratio (log) | -0.01 | -0.03, 0.00 | 0.10 | 0.2 |
| Proportion of standard drinks (log) | -0.01 | -0.04, 0.01 | 0.3 | 0.4 |
| Sex | -0.01 | -0.03, 0.01 | 0.2 | 0.4 |
| Age | 0.00 | -0.01, 0.01 | 0.8 | 0.8 |
| PLE | 0.00 | 0.00, 0.00 | 0.068 | 0.2 |
| ^1^False discovery rate correction for multiple testing | | | | |
| CI = Confidence Interval; PLE = psychotic-like experiences | | | | |

*****Right Thalamus*****

| **Variable** | **Beta** | **95% CI** | **p-value** | **q-value**^1^ |
| --- | --- | --- | --- | --- |
| Cannabis sessions ratio (log) | -0.01 | -0.02, 0.01 | 0.3 | 0.5 |
| Proportion of standard drinks (log) | -0.01 | -0.04, 0.01 | 0.3 | 0.5 |
| Sex | 0.01 | -0.01, 0.02 | 0.5 | 0.6 |
| Age | 0.00 | -0.01, 0.01 | 0.6 | 0.6 |
| PLE | 0.00 | 0.00, 0.00 | 0.036 | 0.2 |
| ^1^False discovery rate correction for multiple testing | | | | |
| CI = Confidence Interval; PLE = psychotic-like experiences | | | | |

*****Ventral Tegmental Area*****

| **Variable** | **Beta** | **95% CI** | **p-value** | **q-value**^1^ |
| --- | --- | --- | --- | --- |
| Cannabis sessions ratio (log) | -0.04 | -0.06, -0.02 | <0.001 | <0.001 |
| Proportion of standard drinks (log) | 0.01 | -0.02, 0.05 | 0.5 | 0.5 |
| Sex | -0.05 | -0.08, -0.03 | <0.001 | <0.001 |
| Age | 0.01 | -0.01, 0.02 | 0.3 | 0.4 |
| PLE | 0.00 | 0.00, 0.00 | 0.3 | 0.4 |
| ^1^False discovery rate correction for multiple testing | | | | |
| CI = Confidence Interval; PLE = psychotic-like experiences | | | | |

**Table S21.** Post-hoc models specifying the link between CUD severity and subcortical 1/nT2* among adolescents with no nicotine use during the Timeline Followback measurement period

*****Left Accumbens*****

| **Variable** | **Beta** | **95% CI** | **p-value** | **q-value**^1^ |
| --- | --- | --- | --- | --- |
| CUD severity | -0.01 | -0.03, 0.01 | 0.2 | 0.5 |
| Proportion of standard drinks (log) | -0.02 | -0.07, 0.03 | 0.4 | 0.7 |
| Sex | 0.00 | -0.04, 0.03 | 0.9 | 0.9 |
| Age | 0.01 | 0.00, 0.03 | 0.11 | 0.5 |
| PLE | 0.00 | 0.00, 0.00 | 0.6 | 0.8 |
| ^1^False discovery rate correction for multiple testing | | | | |
| CI = Confidence Interval; PLE = psychotic-like experiences; CUD = cannabis use disorder | | | | |

*****Right Accumbens*****

| **Variable** | **Beta** | **95% CI** | **p-value** | **q-value**^1^ |
| --- | --- | --- | --- | --- |
| CUD severity | -0.01 | -0.03, 0.01 | 0.3 | 0.7 |
| Proportion of standard drinks (log) | -0.01 | -0.05, 0.03 | 0.7 | 0.7 |
| Sex | 0.01 | -0.02, 0.04 | 0.6 | 0.7 |
| Age | 0.01 | 0.00, 0.02 | 0.2 | 0.7 |
| PLE | 0.00 | 0.00, 0.00 | 0.7 | 0.7 |
| ^1^False discovery rate correction for multiple testing | | | | |
| CI = Confidence Interval; PLE = psychotic-like experiences; CUD = cannabis use disorder | | | | |

*****Left Pallidum*****

| **Variable** | **Beta** | **95% CI** | **p-value** | **q-value**^1^ |
| --- | --- | --- | --- | --- |
| CUD severity | -0.03 | -0.07, 0.01 | 0.13 | 0.3 |
| Proportion of standard drinks (log) | 0.08 | -0.01, 0.18 | 0.068 | 0.3 |
| Sex | -0.01 | -0.07, 0.06 | 0.8 | >0.9 |
| Age | 0.01 | -0.02, 0.04 | 0.6 | >0.9 |
| PLE | 0.00 | 0.00, 0.00 | >0.9 | >0.9 |
| ^1^False discovery rate correction for multiple testing | | | | |
| CI = Confidence Interval; PLE = psychotic-like experiences; CUD = cannabis use disorder | | | | |

*****Right Pallidum*****

| **Variable** | **Beta** | **95% CI** | **p-value** | **q-value**^1^ |
| --- | --- | --- | --- | --- |
| CUD severity | -0.02 | -0.06, 0.02 | 0.3 | 0.5 |
| Proportion of standard drinks (log) | 0.08 | 0.00, 0.17 | 0.058 | 0.3 |
| Sex | 0.01 | -0.05, 0.07 | 0.7 | 0.8 |
| Age | 0.02 | -0.01, 0.05 | 0.3 | 0.5 |
| PLE | 0.00 | 0.00, 0.00 | 0.8 | 0.8 |
| ^1^False discovery rate correction for multiple testing | | | | |
| CI = Confidence Interval; PLE = psychotic-like experiences; CUD = cannabis use disorder | | | | |

*****Left Caudate*****

| **Variable** | **Beta** | **95% CI** | **p-value** | **q-value**^1^ |
| --- | --- | --- | --- | --- |
| CUD severity | -0.02 | -0.03, 0.00 | 0.011 | 0.056 |
| Proportion of standard drinks (log) | 0.01 | -0.02, 0.04 | 0.6 | 0.8 |
| Sex | -0.01 | -0.03, 0.01 | 0.3 | 0.5 |
| Age | 0.01 | 0.00, 0.02 | 0.2 | 0.4 |
| PLE | 0.00 | 0.00, 0.00 | >0.9 | >0.9 |
| ^1^False discovery rate correction for multiple testing | | | | |
| CI = Confidence Interval; PLE = psychotic-like experiences; CUD = cannabis use disorder | | | | |

*****Right Caudate*****

| **Variable** | **Beta** | **95% CI** | **p-value** | **q-value**^1^ |
| --- | --- | --- | --- | --- |
| CUD severity | -0.01 | -0.03, 0.00 | 0.041 | 0.2 |
| Proportion of standard drinks (log) | 0.01 | -0.02, 0.04 | 0.6 | 0.7 |
| Sex | -0.01 | -0.03, 0.02 | 0.6 | 0.7 |
| Age | 0.01 | 0.00, 0.02 | 0.2 | 0.4 |
| PLE | 0.00 | 0.00, 0.00 | 0.8 | 0.8 |
| ^1^False discovery rate correction for multiple testing | | | | |
| CI = Confidence Interval; PLE = psychotic-like experiences; CUD = cannabis use disorder | | | | |

*****Left Putamen*****

| **Variable** | **Beta** | **95% CI** | **p-value** | **q-value**^1^ |
| --- | --- | --- | --- | --- |
| CUD severity | -0.01 | -0.03, 0.00 | 0.024 | 0.12 |
| Proportion of standard drinks (log) | 0.00 | -0.03, 0.03 | >0.9 | >0.9 |
| Sex | -0.01 | -0.03, 0.01 | 0.2 | 0.6 |
| Age | 0.00 | -0.01, 0.01 | 0.5 | 0.8 |
| PLE | 0.00 | 0.00, 0.00 | 0.7 | 0.9 |
| ^1^False discovery rate correction for multiple testing | | | | |
| CI = Confidence Interval; PLE = psychotic-like experiences; CUD = cannabis use disorder | | | | |

*****Right Putamen*****

| **Variable** | **Beta** | **95% CI** | **p-value** | **q-value**^1^ |
| --- | --- | --- | --- | --- |
| CUD severity | 0.00 | -0.02, 0.01 | 0.7 | 0.8 |
| Proportion of standard drinks (log) | 0.01 | -0.02, 0.04 | 0.7 | 0.8 |
| Sex | 0.01 | -0.01, 0.03 | 0.5 | 0.8 |
| Age | 0.00 | -0.01, 0.01 | 0.8 | 0.8 |
| PLE | 0.00 | 0.00, 0.00 | 0.5 | 0.8 |
| ^1^False discovery rate correction for multiple testing | | | | |
| CI = Confidence Interval; PLE = psychotic-like experiences; CUD = cannabis use disorder | | | | |

*****Left Thalamus*****

| **Variable** | **Beta** | **95% CI** | **p-value** | **q-value**^1^ |
| --- | --- | --- | --- | --- |
| CUD severity | 0.00 | -0.01, 0.01 | 0.4 | 0.5 |
| Proportion of standard drinks (log) | -0.02 | -0.04, 0.00 | 0.10 | 0.3 |
| Sex | -0.01 | -0.02, 0.01 | 0.4 | 0.5 |
| Age | 0.00 | -0.01, 0.01 | 0.8 | 0.8 |
| PLE | 0.00 | 0.00, 0.00 | 0.074 | 0.3 |
| ^1^False discovery rate correction for multiple testing | | | | |
| CI = Confidence Interval; PLE = psychotic-like experiences; CUD = cannabis use disorder | | | | |

*****Right Thalamus*****

| **Variable** | **Beta** | **95% CI** | **p-value** | **q-value**^1^ |
| --- | --- | --- | --- | --- |
| CUD severity | 0.00 | -0.01, 0.01 | 0.8 | 0.8 |
| Proportion of standard drinks (log) | -0.02 | -0.04, 0.01 | 0.15 | 0.4 |
| Sex | 0.01 | -0.01, 0.03 | 0.4 | 0.6 |
| Age | 0.00 | -0.01, 0.01 | 0.6 | 0.8 |
| PLE | 0.00 | 0.00, 0.00 | 0.035 | 0.2 |
| ^1^False discovery rate correction for multiple testing | | | | |
| CI = Confidence Interval; PLE = psychotic-like experiences; CUD = cannabis use disorder | | | | |

*****Ventral Tegmental Area*****

| **Variable** | **Beta** | **95% CI** | **p-value** | **q-value**^1^ |
| --- | --- | --- | --- | --- |
| CUD severity | -0.03 | -0.04, -0.01 | <0.001 | <0.001 |
| Proportion of standard drinks (log) | -0.01 | -0.04, 0.03 | 0.7 | 0.7 |
| Sex | -0.05 | -0.07, -0.03 | <0.001 | <0.001 |
| Age | 0.01 | 0.00, 0.02 | 0.15 | 0.2 |
| PLE | 0.00 | 0.00, 0.00 | 0.6 | 0.7 |
| ^1^False discovery rate correction for multiple testing | | | | |
| CI = Confidence Interval; PLE = psychotic-like experiences; CUD = cannabis use disorder | | | | |

Bibliography

1.	Nieto-Castanon A. Handbook of functional connectivity magnetic resonance imaging methods in CONN: Hilbert Press; 2020.

2.	Andersson JLR, Hutton C, Ashburner J, Turner R, Friston K. Modeling Geometric Deformations in EPI Time Series. NeuroImage. 2001;13(5):903-19. doi:<https://doi.org/10.1006/nimg.2001.0746>

3.	Friston KJ, Ashburner J, Frith CD, Poline J-B, Heather JD, Frackowiak RSJ. Spatial registration and normalization of images. Human Brain Mapping. 1995;3(3):165-89. doi:<https://doi.org/10.1002/hbm.460030303>

4.	Sladky R, Friston KJ, Tröstl J, Cunnington R, Moser E, Windischberger C. Slice-timing effects and their correction in functional MRI. NeuroImage. 2011;58(2):588-94. doi:<https://doi.org/10.1016/j.neuroimage.2011.06.078>

5.	Whitfield-Gabrieli S, Nieto-Castanon A, Ghosh S. Artifact detection tools (ART). Cambridge, MA. Release Version. 2011;7(19):11.

6.	Nieto-Castanon A. Preparing fMRI Data for Statistical Analysis. arXiv preprint arXiv:2210.13564. 2022.

7.	Power JD, Mitra A, Laumann TO, Snyder AZ, Schlaggar BL, Petersen SE. Methods to detect, characterize, and remove motion artifact in resting state fMRI. NeuroImage. 2014;84:320-41. doi:<https://doi.org/10.1016/j.neuroimage.2013.08.048>

8.	Calhoun VD, Wager TD, Krishnan A, Rosch KS, Seymour KE, Nebel MB, et al. The impact of T1 versus EPI spatial normalization templates for fMRI data analyses. Human Brain Mapping. 2017;38(11):5331-42. doi:<https://doi.org/10.1002/hbm.23737>

9.	Ashburner J. A fast diffeomorphic image registration algorithm. NeuroImage. 2007;38(1):95-113. doi:<https://doi.org/10.1016/j.neuroimage.2007.07.007>

10.	Ashburner J, Friston KJ. Unified segmentation. NeuroImage. 2005;26(3):839-51. doi:<https://doi.org/10.1016/j.neuroimage.2005.02.018>

11.	Friston KJ, Williams S, Howard R, Frackowiak RSJ, Turner R. Movement-Related effects in fMRI time-series. Magnetic Resonance in Medicine. 1996;35(3):346-55. doi:<https://doi.org/10.1002/mrm.1910350312>

12.	Hallquist MN, Hwang K, Luna B. The nuisance of nuisance regression: Spectral misspecification in a common approach to resting-state fMRI preprocessing reintroduces noise and obscures functional connectivity. NeuroImage. 2013;82:208-25. doi:<https://doi.org/10.1016/j.neuroimage.2013.05.116>
